# MoMih1 is indispensable for asexual development, cell wall integrity, and pathogenicity of *Magnaporthe oryzae*

**DOI:** 10.3389/fpls.2023.1146915

**Published:** 2023-03-14

**Authors:** Shiyi Liu, Xinli Gong, Ji Ma, Shuaishuai Wang, Min Guo

**Affiliations:** ^1^ Key Laboratory of Biology and Sustainable Management of Plant Diseases and Pests of Anhui Higher Education Institutes, Anhui Agricultural University, Hefei, China; ^2^ College of Plant Protection, Anhui Agricultural University, Hefei, China

**Keywords:** *M. oryzae*, dual-specificity phosphatase, *MoMih1*, conidiogenesis, pathogenicity

## Abstract

Asexual spore serves as essential inoculum of rice blast during the disease cycle, and differentiation of young conidium from conidiophore is intimately regulated by cell cycle. Mih1 encodes a dual-specificity phosphatase that involved in the G2/M transition of the mitotic cell cycle by regulating the Cdk1 activity in eukaryotes. Till now, the roles of Mih1 homologue, however, remain unclear in *Magnaporthe oryzae*. We here functionally characterized the Mih1 homologue MoMih1 in *M. oryzae*. MoMih1 is localized to both the cytoplasm and nucleus and can physically interact with the CDK protein MoCdc28 *in vivo*. Loss of MoMih1 led to delayed nucleus division and a high level of Tyr15 phosphorylation of MoCdc28. The *MoMih1* mutants showed retarded mycelial growth with a defective polar growth, less fungal biomass, and shorter distance between diaphragms, compared with the *KU80*. Asexual reproduction altered in *MoMih1* mutants, with both abnormal conidial morphogenesis and decreased conidiation. The *MoMih1* mutants severely attenuated the virulence to host plants due to the impaired ability of penetration and biotrophic growth. The incapability of scavenging of host-derived reactive oxygen species, which was possibly ascribed to the severely decreased extracellular enzymes activities, were partially associated with deficiency of pathogenicity. Besides, the *MoMih1* mutants displayed also improper localization of retromer protein MoVps26 and polarisome component MoSpa2, and defects of cell wall integrity (CWI), melanin pigmentation, chitin synthesis, and hydrophobicity. In conclusion, our results demonstrate that MoMih1 plays pleiotropic roles during fungal development and plant infection of *M. oryzae*.

## Introduction

Rice blast, caused by the fungal pathogen *Magnaporthe oryzae* (synonym to *Pyricularia oryzae*), is one of the most destructive diseases that seriously threatens the rice production worldwide (Wilson and Talbot, 2009). The blast fungus differentiates three-celled pyriform spores as the primary inoculum, and their dispersal is essential for the epidemiology of this disease (Fernandez and Orth, 2018). The blast disease begins at the attachment of those asexual spores to the susceptible rice leaves, and under permitted condition, they germinate and develop a specialized dome-shape appressorium at the tip of germ tube, which helps to rupture the rice leaf cuticle cells (Sabnam and Barman, 2017). After penetration, the invasive hyphae grow biotrophically in the initially infected plant cells and thereafter followed by a necrotrophic phase, which resulted in cell death and appearance of disease lesions on the plants (Ebbole, 2007; Zhang et al., 2016).

The development of both pyriform spores and appressorium is one of the most complicated morphological and physiological events (Zhou et al., 2009; Liu et al., 2010; Oses-Ruiz and Talbot, 2017; Oses-Ruiz et al., 2021). The switches from conidiophore growth to young conidium differentiation, and germ tube to melanized appressorium are intimately linked to cell cycle regulation in *M. oryzae* (Liu et al., 2010; Saunders et al., 2010). During conidiation, two rounds of mitosis and cytokinesis have occurred to develop the mature pyriform conidia, in which each cell contains a single nucleus (Liu et al., 2010; Li et al., 2018). Blocking of Dam1, a subunit of DASH complex involved in cell cycle, delayed the mitotic progression and impaired sporulation under permitted conditions (Shah et al., 2019). Cdc14 is a conserved dual-specificity phosphatases that directly accelerates the cytokinesis during cell division (Meitinger et al., 2012). In *M. oryzae*, the *CDC14* homologue is essential for mitosis and cytokinesis during asexual spore differentiation, and its disruption results in defective mitotic exit, and incorrect septum formation during conidium and appressorium development in *M. oryzae* (Li et al., 2018). Besides, the Pombe Cdc15 homology (PCH) MoCDC15, which is required for cytokinesis by directly regulating the formation of actomyosin ring, has also been identified for asexual spore development and plant infection of *M. oryzae* (Goh et al., 2011). During infection process, appressorium mediated plant infection, which is crucial for the full virulence of *M. oryzae*, is tightly regulated by the cell cycle checkpoint (Saunders et al., 2010). Mutation of two checkpoints such as *NIM1* and *NimA* could prevent the fungal infection of rice plants (Saunders et al., 2010). What’s more, the block of cell cycle by prohibiting S-phase checkpoint or mitotic exit also prevents appressorium-mediated plant infection, further highlighting the essential role of cell cycle regulation in determining the full virulence of *M. oryzae* (Saunders et al., 2010; Oses-Ruiz et al., 2017). Though successful proceeding of cell cycle is critical for the development of asexual spores and infection structures, the precise regulatory mechanism of the cell-cycle check point controlled by the special regulator, have yet to be definitively understood in *M. oryzae*.

Protein phosphorylation and dephosphorylation are two most important post-translational modifications that regulate the cellular processes, such as cell cycle, DNA replication, and gene transcription, and have mainly been regulated by protein kinase and protein phosphatase in eukaryotes (Zhao et al., 2007; Li et al., 2012). Dual-specificity phosphatases are recognized to dephosphorylate tyrosine and serine/threonine residues within the substrate, and thus control a serial of fundamental physiological processes such as cell growth, differentiation, and apoptosis (Tonks, 2006). Mih1 encodes a dual-specificity phosphatase that activates the enzymes activity of cyclin-dependent protein kinase (Cdk1) by removing the phosphate group on the catalytic subunit Cdc28, then promotes G2/M transition of mitotic cell cycle (Camps et al., 2000; Pal et al., 2008). In fungi like *Schizosaccharomyces pombe*, *Ustilago maydis* and *Aspergillus nidulans*, the Cdc25 encodes a dual-specificity protein phosphatase that is essential for the G2/M phase transition, and the disruption of Cdc25 makes the mutant postpone entry into mitosis and develop abnormally large cell (Lew and Kornbluth, 1996; Sgarlata and Perez-Martin, 2005). However, in budding yeast *Saccharomyces cerevisiae*, inactivation of Mih1, a homolog of *S. pombe* Cdc25, could only lead to a moderate delay of mitotic entry, which is inconsistent with the previous findings in other fungi, suggesting a potential exist of redundancy of the dephosphorylation of Cdk1 in different eukaryotes (Pal et al., 2008). In addition, Mih1 has been confirmed to control the phosphorylation state of Vps26, a subunit of retromer subcomplex that directly recognizes the sorting signal in cargo transport, and mutation of Mih1 lead to decreased affinity of retromer to the targets, defective polar growth and pathogenesis of the fungi (Riquelme, 2013; Zheng et al., 2015; Cui et al., 2017; Qu et al., 2022). Taken together, the cell cycle was differentially regulated in the fungi when responses to diverse environmental stimuli, and understanding the roles of dual-specificity phosphatase Cdc25 would provide novel clues to insight of regulatory mechanism of the cell cycle in eukaryotes.

In this study, we report a crucial role for a putative cell division control protein Cdc25 which shows high similarity to the *S. cerevisiae* Mih1 during fungal development and pathogenesis in *M. oryzae*. Our results demonstrate that the loss of *MoMih1* led to delayed nucleus division and a high level of Tyr15 phosphorylation of MoCdc28. The *MoMih1* mutant was severely reduced in polar growth and asexual reproduction. The virulence of *MoMih1* mutants were significantly attenuated due to the impaired ability of penetration and biotrophic growth. The *MoMih1* mutants displayed also improper localization of retromer protein MoVps26 and polarisome component MoSpa2, and defects of cell wall integrity (CWI), melanin pigmentation, chitin distribution, hydrophobicity, and extracellular enzymes activities. Taken together, our results indicate that MoMih1 is an important regulator required for the fungal development and plant infection of *M. oryzae*.

## Materials and methods

### Strains, cultural conditions, and phenotype assays

The wild-type strain Guy11 was used the parental strain to delete the *MoGin4* and *MoCyc2* gene, while the *KU80* strain was used as the parental strain to disrupt the *MoMih1* gene in *M. oryzae* (Villalba et al., 2008). Mycelia used for microscopic examination, DNA and RNA isolation, protein extraction were cultured in liquid CM at 28°C for 24 to 48 h. To assay vegetative growth, mycelial plugs of *KU80*, *MoMih1-41*, *MoMih1-44*, and *MoMih1*c were respectively inoculated on complete medium (CM), minimal medium (MM), oatmeal medium (OM), and rice straw decoction and corn medium (RDC) (Qi et al., 2012), and cultured at 28°C in darkness for 5 d. To measure fungal biomass, four mycelial plugs of tested strains were cultured in 150 mL liquid CM at 28°C for 3 d, then froze dry to measure dry weight.

Conidia suspension of *KU80*, *MoMih1-41*, *MoMih1-44* and *MoMih1*c were dropped on hydrophilic coverslips to develop mycelium, then stained with CFW to examine septum formation. For melanin pigmentation, all strains were cultured on CM for 5 d and/or in liquid CM for 7 d at 28°C in darkness. To investigate asexual reproduction, all strains were cultured on RDC medium, and the conidia were collected, and counted as the reference described (Guo et al., 2017). Conidial morphology and size, and the average numbers of septa per conidia were recorded using a light microscope. To evaluate the responses to different stressors, all strains were inoculated on CM plates supplemented with or without 5 mM H_2_O_2_, 200 μg/mL CFW, 200 μg/mL CongoRed, and 5 mM caffeine, respectively, and then cultured at 28°C for 5 d. To detect CWI, equal amount of mycelia of tested strain was treated with 7.5 mg/mL lysing enzyme, and the release of protoplasts was recorded after enzyme treatment for 30, 60, and 90 min.

### Phylogenetic analysis

To investigate the phylogenetic relationship among the orthologues involved in the cell cycle in different species, the amino acid sequences of each orthologue in *S. cerevisiae* were used as a prey to search the NCBI database, all the orthologous proteins in diverse species were downloaded, and aligned using the Muscle (Edgar, 2004). The aligned sequences were imported into software IQ-TREE 2, and thereafter phylogenetically analyzed to evaluate the evolutionary relationship among those orthologues using the maximum likelihood method (Minh et al., 2020).

### Targeted gene deletion and complementation

The 1200 bp DNA fragments, which respectively flanked the upstream and downstream of the *MoMih1* gene, were amplified from the *M. oryzae* genomic DNA with primers *MoMih1*-1F/*MoMih1*-2R and *MoMih1*-3F/*MoMih1*-4R (**Table S1**). The hygromycin B cassette (*HPH*) was amplified from plasmid pCB1003 with primers *HPH*-F/*HPH*-R (**Table S1**). The above DNA fragments were fused to the enzymes digested (*Xba*I and *Hin*dIII) pKO1B vector (Lu et al., 2014) using the gap repair system in yeast FY834. The resultant plasmid pKO1B-*MoMih1*-*HPH* was transformed into *Agrobacterium tumefaciens* strain AGL1, and the *MoMih1* deletion mutants was generated using the *A. tumefaciens*-mediated transformation (ATMT) of *M. oryzae*. The putative *MoMih1* mutants were further verified by PCR, RT-PCR, and Southern blot.

To generate complemented strain, a 3030 bp DNA fragment containing a native promoter (1180 bp) and a full length of *MoMih1* coding region (1850 bp) was amplified using primers Mih1pF/Mih1pR (**Table S1**) and transformed into yeast strain XK125 together with *Xho*I linearized pYF11. The complemental vector pYF11-*MoMih1* was introduced into the *MoMih1-44* mutant by PEG-mediated transformation of *M. oryzae*. Both *MoGin4* (MGG_02810) and *MoCyc2* (MGG_07065) deletion mutants were generated following the above-described methods.

### Localization of MoMih1 in *M. oryzae*

To observe the localization of MoMih1 in *M. oryzae*, the full length of *MoMih1* coding region (1853 bp) was amplified with primers RP27Mih1pF/Mih1pR (**Table S1**) and ligated with *Xho*I linearized pYF11. The resultant plasmid pYF11-*MoMih1-eGFP* was transformed into Guy11. To visualize the colocalization of MoMih1 with the nucleus signal, the plasmid containing *H1-mRFP* fusion gene was transformed into strain expressing *MoMih1*-e*GFP*. The derivative strains, which expressed *MoMih1*-e*GFP* or both *MoMih1*-e*GFP* and *H1*-*mRFP*, were examined under a confocal laser scanning microscope (CLSM).

### Localization of MoVps26 and MoSpa2 in *MoMih1* mutant

The full length of *MoVps26* coding region (1098 bp) was amplified from genomic DNA of Guy11 with primers Vps26pF/Vps26pR (**Table S1**), and then ligated to *Xho*I digested pYF11 following the above-described procedures. The plasmid pYF11-*MoVps26-eGFP* was respectively introduced in *KU80* and *MoMih1-44* mutant, and then hyphal tips of each strain expressing MoVps26-eGFP fusion protein were examined under CLSM. To evaluate whether the microtubule-destabilizing drug affected the localization of MoVps26-eGFP, the above transformants were cultured in liquid CM with or without 0.4 μg/mL benomyl, and then hyphal tips of them were examined under CLSM.

To determine the localization of MoSpa2 in *MoMih1* mutant, the full length of *MoSpa2* coding region (3.0 kb) was cloned with primers Spa2pF/Spa2pR (**Table S1**) and ligated into pYF11 as the procedures described above. The plasmid pYF11-*MoSpa2-eGFP* was then introduced into *KU80* and *MoMih1-44* mutant, respectively, and hyphal tips of the derivative strain expressing MoSpa2-eGFP were examined under CLSM.

### Plant infection

To test pathogenicity, conidial suspensions (1 × 10^4^ spores/mL) of *KU80*, *MoMih1-41*, *MoMih1-44* and *MoMih1*c were dropped on barley leaves (*Hordeum vulgare* cv Golden Promise; one-week-old), and/or sprayed on rice seedlings (*Oryza sativa* cv CO-39; two-week-old), respectively. The fungi-inoculated plants were placed in a moist chamber at 28°C in dark for 24 h, and then shifted to a photoperiod of 12 h light for another 96 h. To examine fungal infection, conidial suspension (1 × 10^4^ spores/mL) was dropped on lower epidermis of barley leaves, cultured at 28°C for 24 h and/or 48 h, and then invasive hyphae were viewed under light microscopy. The DAB staining was performed following the previous methods (Guo et al., 2017).

### Chitin content determination

The chitin contents of *KU80*, *MoMih1-41*, *MoMih1-44* and *MoMih1*c were determined following the previous method (Yin et al., 2016). 5 mg freeze-dried fungal powder was mixed with 1 mL 6% KOH solution, water bathed at 80°C for 90 min, and then centrifuged (16 000g, 10 min). The sediment was washed by 1 mL PBS for three times, resuspended in 0.5 mL Mcilvine’s buffer (pH 6), and then added 100 μL chitinase (C6137, Sigma-Aldrich, Shanghai, China) in suspension. After reacting in water bath at 37°C for 16 h, 100 μL sodium borate solution (0.27 M, pH 9) was added in the tubes, then boiled at 100°C for 10 min. 1 mL diluted Ehrlich’s reagent solution (10-folds) was finally added to the reaction tubes, and then incubated at 37°C for 20 min. The absorbance value of each sample was determined at OD_585_. A standard curve was prepared using the D-GlcNAc (A8625, Sigma-Aldrich, Shanghai, China). This experiment was repeated three times.

### Extracellular enzyme activities assay

The strains of *KU80*, *MoMih1-41*, *MoMih1-44* and *MoMih1*c were respectively inoculated on CM plates with or without 0.2 mM ABTS (2,2’-Azinobis-(3-ethylbenzthiazoline-6-sulphonate)), placed in chamber at 28°C for 24 h, and then recorded the purple hydrolysis circle on the media. In liquid CM, extracellular enzyme activities, including the laccases and peroxidases, were determined following the previous procedures (Chi et al., 2009). These experiments were repeated three times.

### Quantitative real-time PCR

The total RNA of each sample was extracted and reversely transcribed following the previous procedures (Guo et al., 2016). The expression of target genes involving in melanogenesis, conidiation, hydrophobicity or genes encoding peroxidase, laccase, chitin synthase were detected using the BIO-RAD CFX96 touch qRT-PCR system (Bio-Rad, Hercules, California, USA) according to the established procedures (Guo et al., 2016). All the primers used here are listed in **Table S1**.

### Assays for nuclear distribution and autophagy

To determine the roles of MoMih1 on cytokinesis, the vector pMoC^H245Y^-*H3-mRFP* was respectively transformed in *KU80* and *MoMih1-44* mutant *via* ATMT to generate *KU80*-*H3*-*mRFP* and *MoMih1*-*H3*-*mRFP* strains. To visualize nuclear distribution, both mycelia and conidia of each tested strain were cultured as described above, stained with CFW, and then visualized by CLSM.

To determine the roles of MoMih1 during autophagy, the vector pMoC^H245Y^-*eGFP*-*ATG8* was transformed into *KU80* and *MoMih1-44* mutant, respectively. The derivative strains of *KU80*-*eGFP*-*ATG8* and *MoMih1*-*eGFP*-*ATG8* were respectively grown in liquid CM at 28°C for 36 h, then transferred to nitrogen starvation (SD-N) medium for another 2 or 4 h. The eGFP-MoAtg8 fusion protein and free GFP were detected using the anti-GFP antibodies, and analyzed by the ImageJ software to calculate the ratio of free GFP and fusion eGFP-MoATG8 (Zhu et al., 2018).

### Yeast two-hybrid assay

The full-length of the coding sequences of *MoMih1* and *MoCdc28* were cloned, and inserted into the bait construct pGADT7 (630442, Clontech, Dalian, China) and prey construct pGBKT7 (630443, Clontech, Dalian, China), respectively. Both the resultant bait construct pGADT7-*MoMih1* and prey construct pGBKT7-*MoCdc28* were co-transformed into yeast strain AH109 (MATα, *trp1-901*, *leu2-3*, 112, *ura3-52*, *his3-200*), and proteins interaction was validated following the described methods (Zheng et al., 2021a).

### BiFC assay

The *MoMih1*-^C^YFP and *MoCdc28*-^N^YFP fusion constructs were generated by ligating the coding sequences of *MoMih1* and *MoCdc28*, which were amplified with primers pHZ68Mih1F/pHZ68Mih1R and pHZ65Cdc28F/pHZ65Cdc28, into pHZ68 and pHZ65, respectively. The vector pairs of *MoMih1*-^C^YFP and *MoCdc28*-^N^YFP, *MoMih1*-^C^YFP and ^N^YFP, ^C^YFP and *MoCdc28*-^N^YFP, and ^C^YFP and ^N^YFP were simultaneously transformed into Guy11. YFP signals were examined under the CLSM. The primers are listed in **Table S1**.

### Assay for Cdc28 phosphorylation

To determine the phosphorylation level of Cdc28, total protein of the tested strains was separated by 12% SDS-PAGE and immunoblotted with an anti-phospho-Cdc2 (Tyr^15^) antibody (Cell Signaling Technology). The anti-Cdc2 antibody (Santa Cruz Biotechnology) was used as a negative control.

## Results

### Identification and deletion of *MoMih1*, *MoGin4* and *MoCyc2* gene in *M. oryzae*

**Figure 1:**
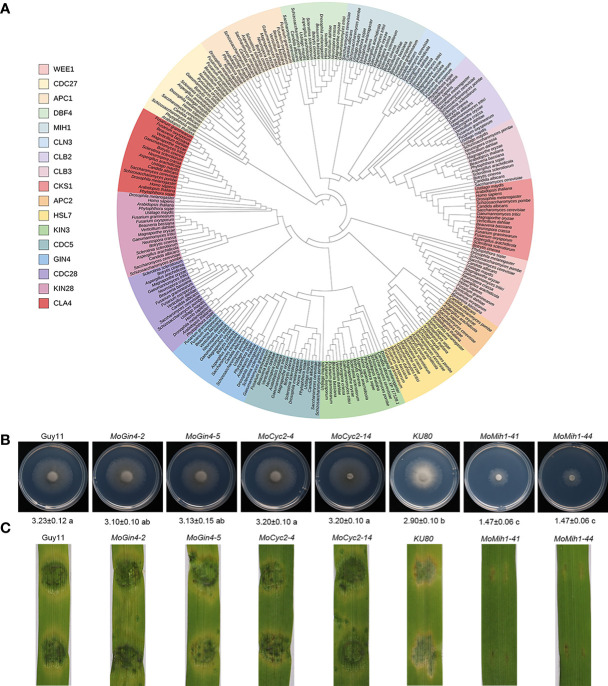
The orthologues of cell cycle and morphogenetic proteins in *M. oryzae*. **(A)** Phylogenetic analysis and classification of cell cycle and morphogenetic related genes in diverse species. IQ-TREE 2 was used to establish a phylogenetic tree based on the full length of these proteins using the maximum likelihood method. **(B)** The mycelial growth of the tested strains. The colony diameters of the tested strains were assayed on MM medium at 5 dpi. **(C)** The pathogenic assay of the tested strains. Mycelial plugs of the tested strains were inoculated on 7-day-old barley leaves, and the diseased leaves were recorded at 5 dpi.

The proceeding of cell cycle is essential for the precise initiation of conidiophore and appressorium, and is accurately regulated by the cell cycle-related genes in filamentous fungus (Saunders et al., 2010; Oses-Ruiz and Talbot, 2017; Yue et al., 2017). In this study, a family of cell cycle-related proteins was identified and phylogenetically grouped into 17 clades (**Figure 1A**). Previously, two cell cycle-related genes, including *MoCks1* (Cks1 family) and *CHM1* (CLA4 family), were individually deleted and functionally characterized in *M. oryzae* (Li et al., 2004; Yue et al., 2017). In this study, to investigate the roles of the rest 15 cell cycle-related genes in *M. oryzae*, a high-throughput gene deletion system (Lu et al., 2014) was used following the homologous gene disruption strategy (**Figure S1A**). At present, three of them, including *MoMih1* (MGG_07734), *MoGin4* (MGG_02810) and *MoCyc2* (MGG_07065), were individually deleted from the parental strain *KU80* or Guy11, respectively, and validated by PCR, RT-PCR and Southern blot (**Figures S1B–D**; **Figures S2A–D**). The two mutants (*MoGin4* and *MoCyc2*), except for *MoMih1*, were dispensable for vegetative growth and pathogenicity, and thus did not perform further evaluations (**Figures 1B, C**). Subsequent bioinformatic analysis revealed that MoMih1 encodes a dual-specificity phosphatase that contains a conserved DCR motif (aa 392-394) and HCXXXXXR sequence (aa 434-441) (**Figure S3**) unique to Cdc25 and Mih1 phosphatases in *S. pombe* and *S. cerevisiae*, respectively, implying a conserved role of MoMih1 in the cell cycle regulation during the development and plant infection of *M. oryzae*.

### Subcellular localization of MoMih1 in *M. oryzae*

**Figure 2:**
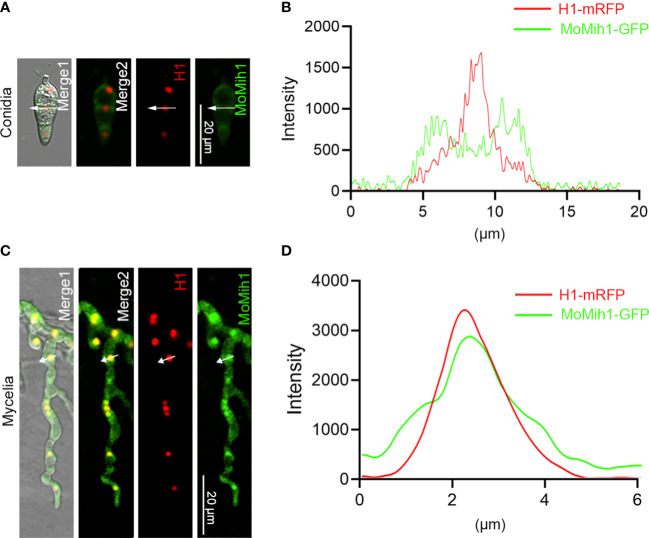
The subcellular localization of MoMih1 in *M. oryzae*. **(A)** The conidia of the transformants co-expressing the MoMih1-eGFP and H1-mRFP fusion protein were examined under a CLSM. Bar = 20 μm. **(B)** Line scan graph analysis of the fluorescence. Line scan graph indicates that MoMih1 don’t colocalized with the histone protein at spore stage. **(C)** The hyphae of the transformants co-expressing the MoMih1-eGFP and H1-mRFP fusion protein were examined under a CLSM. Bar = 20 μm. **(D)** Line scan graph analysis of the red and green fluorescence. Line scan graph analysis of fluorescence indicates the co-localization of MoMih1 with histone at mycelia.

To ascertain the cellular localization of MoMih1 in *M. oryzae*, the MoMih1-eGFP fusion protein was transformed into the wild type strain Guy11, and then examined under CLSM. The MoMih1-eGFP fusion protein was mostly distributed in cytoplasm of the conidia (**Figures 2A, B**; **Figure S4A**), and mainly concentrated as patches in the vegetative and invasive hyphae (**Figures S4B, C**). To ascertain whether the patches of MoMih1-eGFP was localized at nucleus in *M. oryzae*, the red fluorescent fusion protein H1-RFP was co-expressed in the *MoMih1-eGFP* strain. A co-localization of MoMih1-eGFP and H1-RFP was observed in the vegetative hyphae of *M. oryzae* (**Figures 2C, D**).

### MoMih1 is required for vegetative growth of *M. oryzae*

**Figure 3:**
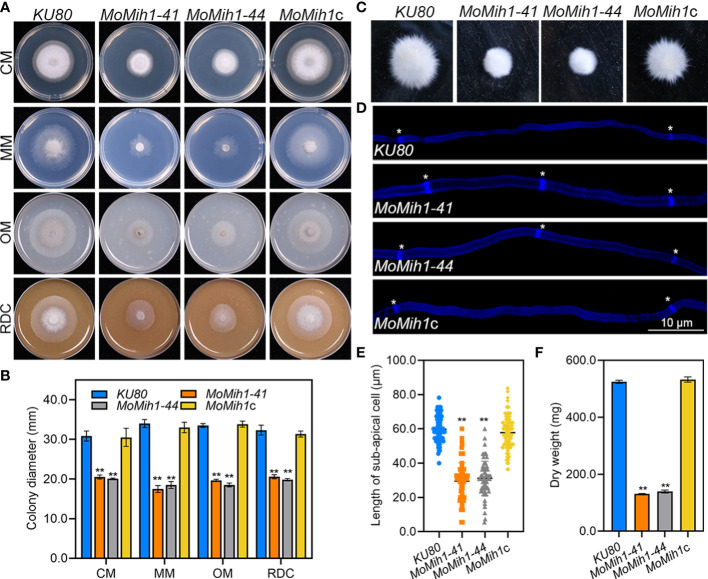
*MoMih1* is required for vegetative growth of *M. oryzae*. **(A)** *MoMih1* mutants displayed retarded growth on four artificial media. The mycelial plugs of *KU80*, *MoMih1-41*, *MoMih1-44*, and *MoMih1*c were inoculated on CM, MM, OM, and RDC, respectively, and cultured at 28°C in darkness for 5 days. **(B)** The analysis of colony diameter of the *MoMih1* mutants. The colony diameter of the tested strains grown on four artificial media were measured at 5 dpi, and then statistically analyzed. The error bars represent the SD of three replicates, whereas “**” represents a significant difference (*p* < 0.01). **(C)** Mycelia growth of the *MoMih1* mutants in liquid CM. All strains were inoculated in liquid CM at 28°C for 48 h in darkness and then photographed. **(D)** Examination of hyphal septa in the *MoMih1* mutants. The mycelia of the tested strains were stained with CFW, and then observed under fluorescent microscopy (UV 330-380 nm). Bar = 10 μm. **(E)** Analysis of the cell length between two diaphragms in vegetative hyphae. The length between two diaphragms of vegetative hyphae of the tested strains were recorded, and statistically analyzed. “**” represents the significant difference between the *KU80* and *MoMih1* mutants (*p* < 0.01). **(F)** Measurement of fungal biomass. Fungal mycelia of *KU80*, *MoMih1-41*, *MoMih1-44*, and *MoMih1*c was collected at 3 dpi, freezing dried, and statistically analyzed (*p* < 0.01).

To validate the biological role of MoMih1 in *M. oryzae*, a complemental strain *MoMih1*c was subsequently generated by reintroducing the full length of *MoMih1* into *MoMih1-44* mutant, and verified by RT-PCR (**Figure S1C**). The mycelial growth rate of *MoMih1* mutants were significantly reduced in comparison to the *KU80* and *MoMih1*c (**Figures 3A, B**). In liquid CM, mycelial pellets of *MoMih1* mutants were much smaller but denser than that of *KU80* and *MoMih1*c (**Figure 3C**). The average length of sub-apical cell of the *MoMih1* mutants was approximately 50% shorter than that of *KU80* and *MoMih1*c (**Figures 3D, E**). Besides, the *MoMih1* mutants significantly decreased the fungal biomass compared with that of *KU80* and *MoMih1*c (**Figure 3F**). These results indicate that MoMih1 is required for the vegetative growth of *M. oryzae*.

### MoMih1 is required for conidiation and conidial morphology of *M. oryzae*

**Figure 4:**
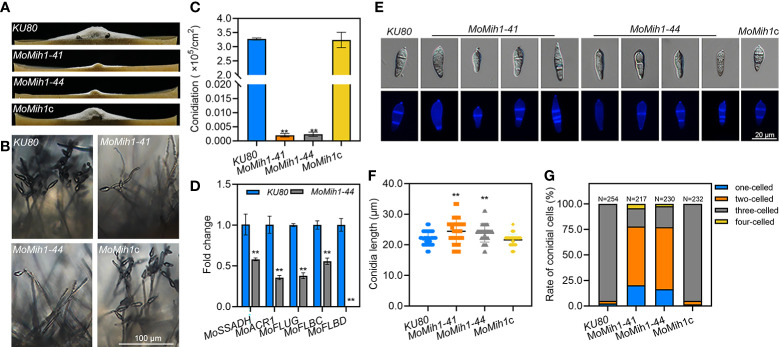
*MoMih1* is required for the conidiation and conidial morphology of *M. oryzae*. **(A)** Comparison of aerial hyphae growth. All strains were grown on RDC media under darkness, and then photographed at 5 dpi. **(B)** Conidia formation on the conidiophores. Light microscopic observation was carried out to examine the conidia development on the conidiophores of the *MoMih1* mutants that grown on RDC at 7dpi. Bar = 100 μm. **(C)** Statistical analysis of conidia reproduction. The conidia formed by each tested strain were collected and statically analyzed. “**” means significant difference among the tested strains (*p* < 0.01), and error bars indicate SD. **(D)** Transcription of the conidiation-related genes. The expression of conidiation-related genes was examined by qRT-PCR in the *KU80* and *MoMih1* mutants. “**” means significant differences (*p*<0.01), and error bars represent SD. **(E)** Conidial morphology of *MoMih1* mutant. Conidia of each tested strain were stained with CFW, and then observed by differential interference contrast (DIC) microscopy and UV fluorescence microscopy. **(F)** Statistical analysis of conidial length. The conidial length was determined by measuring more than 200 conidia, and then statistically analyzed. “**” means significant difference among the strains (*p* < 0.01), and error bars represent SD. **(G)** Ratio of conidia with different number of cells in the *MoMih1* mutant. The proportion of conidia with different cell numbers was statistically analyzed from three independent experiments. “**” means significant difference among the tested strains (*p* < 0.01), and error bars represent SD.

The aerial hyphae growth and conidiophore development were compared, and the *MoMih1* mutants developed less fluffy aerial hyphae than that of *KU80* and *MoMih1*c (**Figure 4A**). Microscopic examination showed that most of the mutants develop abnormal conidiophores, bearing significantly less conidia than that of *KU80* and *MoMih1*c (**Figure 4B**). The conidial production of *MoMih1* mutants was apparently decreased compared with *KU80* and *MoMih1*c (**Figure 4C**). To further understand the potential role of MoMih1 on conidiation, the expression of sporulation-related genes was investigated, and all of them significantly down-regulated in the *MoMih1* mutant, compared with that in *KU80* strain (**Figure 4D**). Besides, the *MoMih1* mutants showed abnormal conidial morphology (**Figure 4E**), with evidently increased conidial length, compared with *KU80* and *MoMih1*c (**Figure 4F**). Meanwhile, the *MoMih1* mutants developed a greater number of one or two-celled conidia, compared with that by *KU80* and *MoMih1*c (**Figure 4G**).

### MoMih1 is required for the pathogenicity of *M. oryzae*

**Figure 5:**
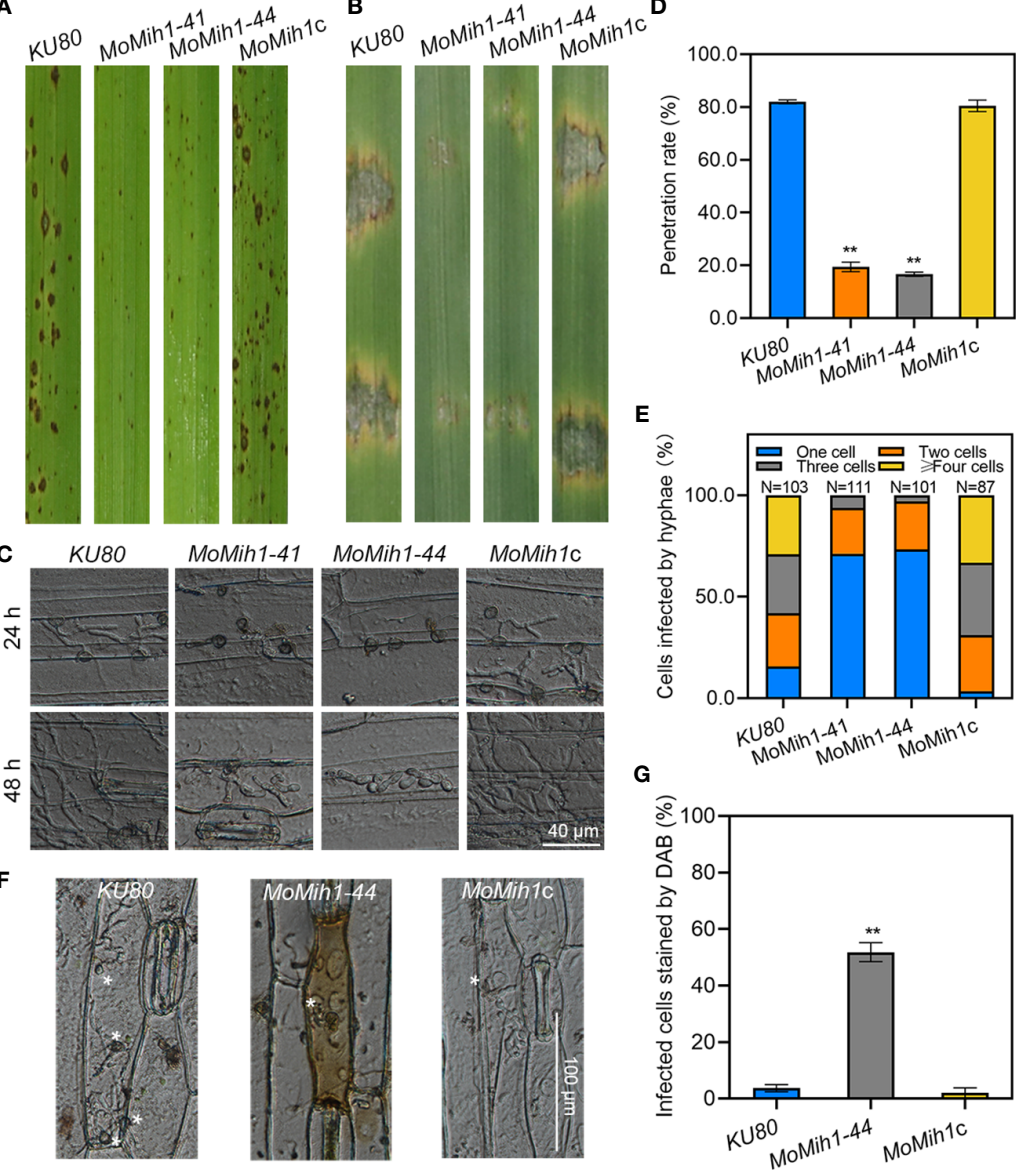
*MoMih1* is essential for pathogenicity of *M. oryzae*. **(A)** Deletion of *MoMih1* affects the virulence of *M. oryzae* on rice leaves. Conidia suspension of the *MoMih1* mutants were sprayed on the rice seedlings, and the diseased leaves were collected at 5 dpi. **(B)** Pathogenic assays on barley leaves. Conidial suspension of *KU80*, *MoMih1* mutants and *MoMih1*c was inoculated on barley leaves, and the diseased leaves were collected at 5 dpi. **(C)** Penetration assay. Conidial suspension of *KU80*, *MoMih1* mutants and *MoMih1*c was inoculated on the barley leaves, cultured under the condition of 28°C for 24 h or 48 h, and then examined under light microscopy. **(D)** Statistical analysis of penetration rate. The appressoria mediated penetration of barley epidermal cells were examined, measured at 24 hpi, and then statistically analyzed. “**” represents significant difference between *KU80* and *MoMih1* mutants (*p* < 0.01), and error bars represent SD. **(E)** The *MoMih1* deletion affects the invasive hyphae expansion. The percentage of plant cells occupied by the invasive hyphae of each strain was measured and analyzed at 48 hpi. **(F)** The *MoMih1* deletion resulted in the accumulation of ROS at infection sites. Conidial suspensions (1 × 10^4^ spores mL^-1^) of *KU80*, *MoMih1-44* mutant and *MoMih1*c were inoculated on barley leaves for 24 h, and then stained with DAB solution. Plant cells infected by the *MoMih1* mutant was mostly stained with DAB. Bar = 100 μm. **(G)** Statistical analysis of the proportion of plant cells stained by DAB. “**” means significant difference between *KU80* and *MoMih1* mutants (*p* < 0.01), and error bars represent SD.

The pathogenicity of *KU80*, *MoMih1* mutants and *MoMih1*c was conducted on both rice and barley leaves. The *MoMih1* mutants significantly attenuated the virulence on host plant leaves, compared with *KU80* and *MoMih1*c (**Figures 5A, B**). The infection ability of the *MoMih1* mutants was impaired, with most of them exhibiting incapability of rupturing the barley leaf epidermis cells, compared with *KU80* and *MoMih1*c (**Figure 5C**). The penetration rate of *MoMih1* mutants was approximately 60% less than that of by *KU80* and *MoMih1*c (**Figure 5D**). At 48 hpi, the invasive hyphae of the *MoMih1* mutants were mostly restricted in the initially invaded cells, in contrast to the free expanding growth to the neighboring cells by the *KU80* and *MoMih1*c (**Figures 5C, E**), suggesting that MoMih1 plays an important role in the pathogenesis of *M. oryzae*.

### MoMih1 is required for scavenging of host-derived ROS during infection

Successful removal of host-derived reactive oxygen species (ROS) from plant infection site is an essential prerequisite to ensure the infection of *M. oryzae* (Guo et al., 2010; Guo et al., 2011; Wang et al., 2017). The sensitivity of the *MoMih1* mutants to hydrogen peroxide (H_2_O_2_) was firstly verified, with all of them showing incremental sensitivity to H_2_O_2_, compared to *KU80* and *MoMih1*c (**Figures S5A, B**). Since the *MoMih1* mutants developed a great amount of tiny necrotic lesions on the rice leaves (**Figure 5A**), the ROS burst at the infection site was thus monitored among the tested strains. An average 51.80 ± 3.34% of the plant cells infected by *MoMih1* mutant could be stained by DAB, compared with 3.71 ± 1.28% and 2.00 ± 1.79% of the *KU80* and *MoMih1*c, respectively (**Figures 5F, G**), suggesting the critical role of MoMih1 in detoxifying the host-derived ROS during plant infection. The potential reason for decreased ability to scavenge host-derived ROS was thereafter explored, and the extracellular enzyme activity, including peroxidases and laccases, was almost abolished in *MoMih1* mutants, compared with *KU80* and *MoMih1*c (**Figures S5C, D**). The transcriptions of five putative peroxidase and laccase encoding genes were significantly down regulated in the *MoMih1* mutants, compared with *KU80* (**Figure S5E**).

### MoMih1 is required for CWI in *M. oryzae*

**Figure 6:**
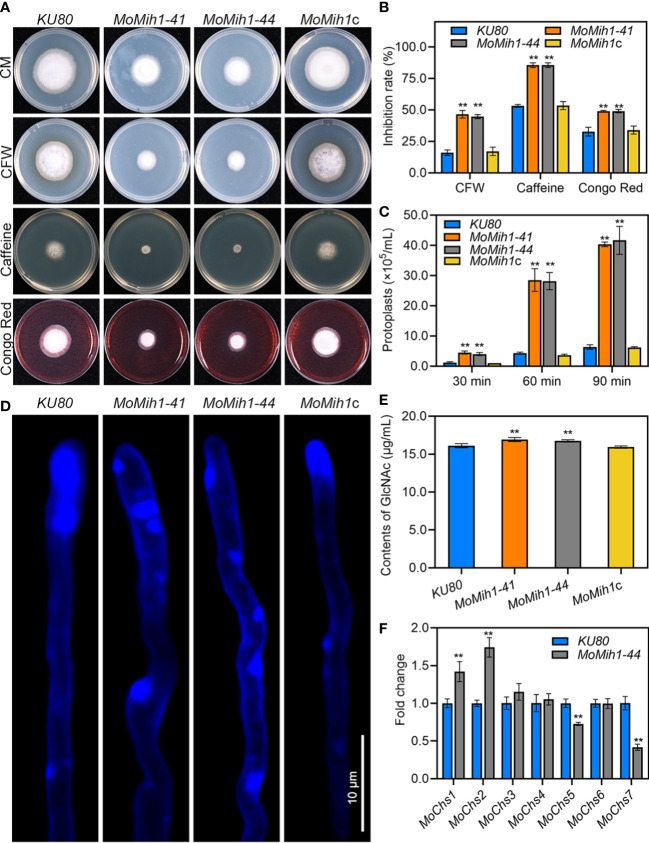
MoMih1 is required for cell wall integrity in *M. oryzae* **(A)** Mycelial growth under cell wall stressors. The *KU80*, *MoMih1* mutants and *MoMih1*c were inoculated on CM with CFW, caffeine, or Congo red, cultured at 28°C for 5 days, and then photographed. **(B)** Statistical analysis of the inhibition rate under different cell wall stressors. “**” mean significant difference between *KU80* and *MoMih1* mutants (*p* < 0.01), and error bars represent SD. **(C)** Protoplasts released under the treatment of cell wall lysing enzyme. The release of protoplast was quantified at 30 min intervals. “**” means significant difference between *KU80* and *MoMih1* mutants (*p* < 0.01), and error bars represent SD. **(D)** Examination of chitin on the cell wall of *MoMih1* mutant. Hypha of *KU80*, *MoMih1* mutants and *MoMih1*c were stained with CFW for 5 min, and then examined under UV fluorescent microscope. Bar = 10 μm. **(E)** Measurement of GlcNac. GlcNac was determined in the *KU80*, *MoMih1* mutants and *MoMih1*c by the fluorimetric Morgan-Elson method. “**” means significant difference between *KU80* and *MoMih1* mutants (*p* < 0.01), and error bars represent SD. **(F)** Expression of chitin synthase encoding genes. Transcription of the chitin synthase encoding genes was compared between *KU80* and *MoMih1* mutants by qRT-PCR. “**” means significant difference between *KU80* and *MoMih1* mutants (*p* < 0.01), and error bars represent SD.

Intact cell wall of *M. oryzae* is a guarantee for the proper infection on rice plants (Feng et al., 2021), the sensitivity of *MoMih1* mutants to different cell wall stressors was therefore tested. The *MoMih1* mutants are more sensitive to those stressors than that of *KU80* and *MoMih1*c (**Figures 6A, B**). Besides, the protoplasts released by the *MoMih1* mutants were also significantly increased compared with *KU80* and *MoMih1*c at each given time (**Figure 6C**), implying a severe defect of CWI in *MoMih1* mutants.

Chitin, one of the main components of fungal cell wall, is essential for the CWI of *M. oryzae* (Yin et al., 2016; Guo et al., 2017). After staining with CFW, the blue fluorescence was mostly congregated at the hyphal tip of *KU80* and *MoMih1*c, however, patches of bright fluorescence were unevenly distributed on the hyphal wall but not the tips of the *MoMih1* mutants (**Figure 6D**). Quantitative detection of chitin contents showed, interestingly, that chitin contents of the *MoMih1* mutant were significantly increased, compared with that of *KU80* and *MoMih1*c (**Figure 6E**). The expression of seven chitin synthase genes was assayed, with *MoChs1* and *MoChs2* showing increased expression, but *MoChs5* and *MoChs7* displaying decreased expression, compared with that of in *KU80* (**Figure 6F**).

### MoMih1 is essential for pigmentation and hydrophobicity in *M. oryzae*

**Figure 7:**
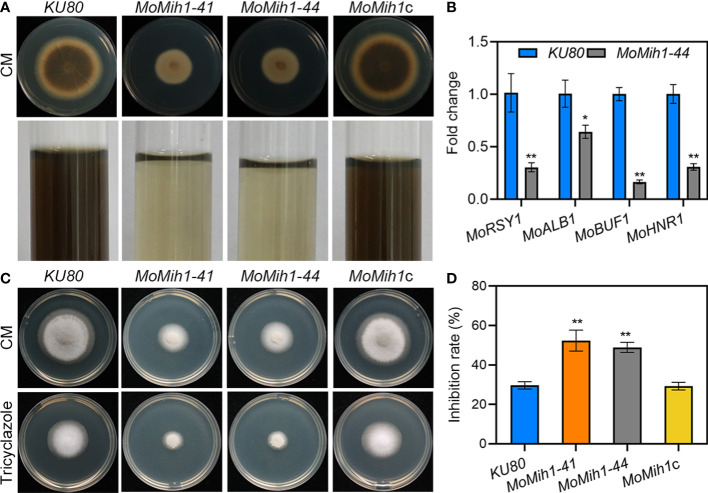
*MoMih1* is required for melanin pigmentation of *M. oryzae*. **(A)** Colony pigmentation of the *MoMih1* mutant. All the strains were inoculated on CM and cultured at 28°C for 5 days. Reduced melanin pigmentation was observed on both CM agar and liquid CM. **(B)** The transcription of melanin synthesis genes in the hypha of *MoMih1* mutant. The expression levels of *MoRSY1*, *MoALB1*, *MoBUF1* and *MoHNR1* was examined by qRT-PCR, with all of them significantly decreased expression in the *MoMih1* mutant. “**” mean significant difference in gene expression between two strains (*p* < 0.01), and error bars represent SD. **(C)** Sensitivity of the *MoMih1* mutant to tricyclazole. The *MoMih1* mutant was inoculated on CM amended with tricyclazole, cultured at 28°C for 5 days, and then fungal colony was recorded. **(D)** Statistical analysis of the inhibition rate under tricyclazole stress. “**” stands for the significant difference between *KU80* and *MoMih1* mutants (*p* < 0.01), and error bars represent SD.

The *MoMih1* mutants were defective in melanin pigmentation compared with *KU80* and *MoMih1*c (**Figure 7A**). The melanin biosynthesis related genes, such as *MoRSY1*, *MoALB1*, *MoBUF1* and *MoHNR1*, were significantly downregulated in the *MoMih1* mutants compared with that in *KU80* (**Figure 7B**). Moreover, the *MoMih1* mutants were more sensitive to tricyclazole, a specific inhibitor of melanin biosynthesis (**Figures 7C, D**), implying that MoMih1 was required for melanin biosynthesis in *M. oryzae*.

The hydrophobicity was determined on the fungal culture of *KU80* and *MoMih1* mutants, respectively. The water droplets remained on the mycelial culture of *KU80* and *MoMih1*c after 24 hpi, in contrast to the immediate soaking into the mycelial cultures by the *MoMih1* mutants (**Figure S6A**). Due to the wettable phenotype of the mutants, the transcription of hydrophobin-encoding genes including *MPG1*, *MHP1*, and two *MHP1* homologs (MGG_09134 and MGG_10105) was verified, and all of them showed remarkably down-regulated expression in *MoMih1* mutant, compared with that of *KU80* (**Figure S6B**).

### MoMih1 is required for polarized growth in *M. oryzae*

**Figure 8:**
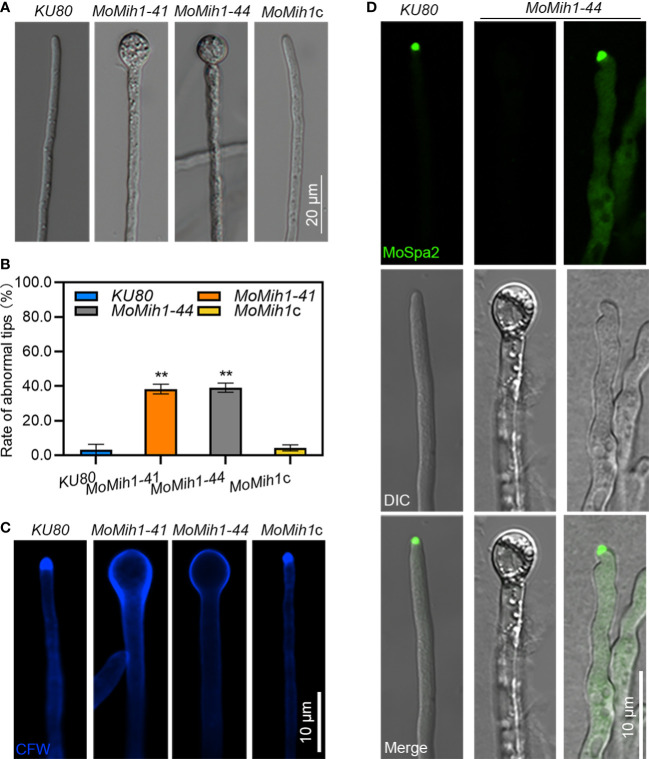
*MoMih1* is required for polarized growth of *M. oryzae* **(A)** Deletion of *MoMih1* resulted in the development of swollen structures at the mycelia tips. All tested strains were inoculated in liquid CM at 28°C for 48 h in darkness, and then swollen structures at the mycelia tips were photographed Bar = 20 μm. **(B)** Statistical analysis of abnormal structures at the mycelial tips. The rate of abnormal mycelial tips was recorded and statistically analyzed. The error bars represent the SD of three replicates, whereas “**” represents a significant difference (*p* < 0.01). **(C)** Chitin distribution on the cell wall of mycelial tips. Hyphal tips of the tested strains were stained by CFW and observed by confocal microscopy. Chitin distribution was abnormal at the swollen tips of the *MoMih1* mutant. Bar = 20 μm. **(D)** Localization of MoSpa2-eGFP in the *MoMih1* mutant. Hyphal tips of *KU80* and *MoMih1-44* that expressed MoSpa2-eGFP were examined by confocal microscopy. Localization of MoSpa2-eGFP to the polarisome was absent in swollen structures of the *MoMih1* mutants. Bar = 20 μm.

In view of the retarded growth of the *MoMih1* mutants, the polar growth was thus examined. Approximate 40% mutant mycelia displayed abnormally swollen tips, compared with *KU80* (**Figures 8A, B**). When stained with CFW, the blue fluorescence was mostly converged at the mycelial tips of *KU80* and *MoMih1*c, but not the swollen tips of *MoMih1* mutants (**Figure 8C**), implying an inactive chitin synthesis in the mycelial tips of *MoMih1* mutant. Spa2 is a component of the polarisome and functions in regulating hyphal morphology and extension during fungal polar growth (Virag and Harris, 2006), therefore, the MoSpa2-eGFP fusion protein were examined in *KU80* and *MoMih1-44*, respectively. The MoSpa2 can accurately localize to the normal growing hyphal tip of *KU80*, but not the swollen tips of the *MoMih1* mutant (**Figure 8D**), suggesting MoMih1 is required for polarisome formation during the polarized growth of *M. oryzae*.

### MoMih1 is required for subcellular localization of MoVps26 in *M. oryzae*

**Figure 9:**
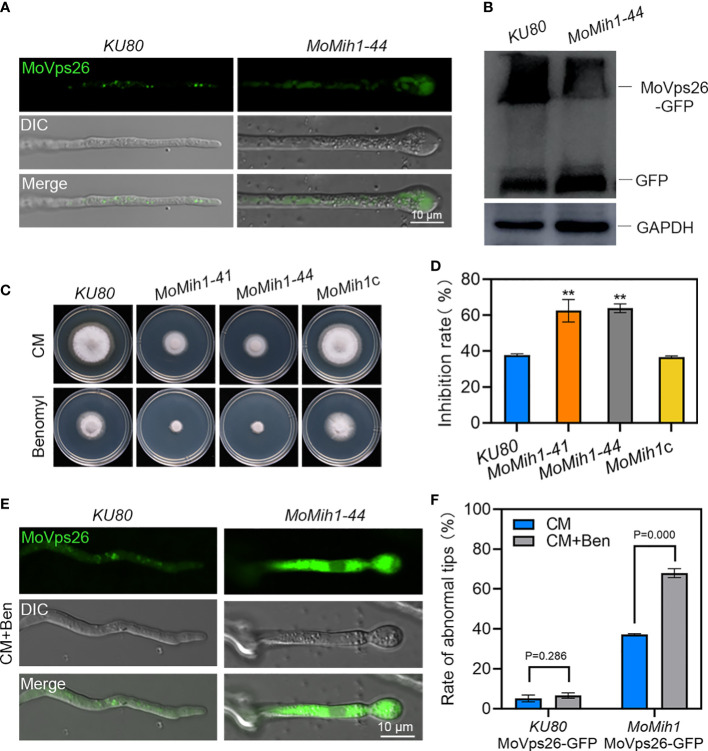
*MoMih1* is required for the localization of MoVps26 in *M. oryzae*. **(A)** The localization of MoVps26-eGFP in *MoMih1* mutants. The strains *KU80* and *MoMih1-44* were respectively expressed the MoVps26-eGFP fusion protein, and then hyphal tips of resultant transformants were examined under confocal microscopy. The localization of MoVps26-eGFP was abnormal in the swollen tips of the *MoMih1* mutants. Bar = 10 μm. **(B)** The proteolysis of MoVps26-eGFP has increased in the *MoMih1* mutant. Mycelia of *KU80* and *MoMih1-44* expressing MoVps26-eGFP were cultured in liquid CM at 28°C for 36 h, and the MoVps26-eGFP fusion protein was detected by immunoblotting with anti-GFP. The blotting of GAPDH was used as a control. **(C)** Mycelia growth under benomyl treatment. All tested strains were inoculated on CM supplemented with 0.4 μg/mL benomyl and cultured at 28 °C for 5 days. **(D)** Statistical analysis of the inhibition rate under benomyl treatment. “**” represents the significant difference between *KU80* and *MoMih1* mutants (p < 0.01), and Error bars represent SD. **(E)** Localization of MoVps26-eGFP under the treatment with 0.4 μg/mL benomyl. The strain *MoMih1*/MoVps26-eGFP was inoculated in liquid CM at 28°C for 24 h in darkness, then added with benomyl and grew at 28°C for 16 h in darkness. Bar = 10 μm. **(F)** Treatment of benomyl stimulated the formation of swollen structures in the *MoMih1* mutants. The swollen structures of the *KU80*/MoVps26-eGFP and *MoMih1*/MoVps26-eGFP was recorded by amending with or without benomyl, and the rate of abnormal mycelial tips was statistically analyzed. The error bars represent the SD of three replicates, whereas “**” represents a significant difference (*p* < 0.01).

In *S. cerevisiae*, Mih1 directly regulates the phosphorylation of Vps26, a subunit of the retromer subcomplex, and thereby modulates the binding affinities of the retromer to specific cargo (Cui et al., 2017), we thus examined the cellular location of MoVps26 in the *MoMih1* mutant. A dispersive distribution of the MoVps26-eGFP was observed in the hyphal cytoplasm of *MoMih1* mutant, compared with the mostly punctate structures in strain *KU80* (**Figure 9A**). The immunoblotting with anti-GFP showed that the proteolysis of MoVps26-eGFP has increased in the *MoMih1* mutant (**Figure 9B**). The mobility of MoVps26-eGFP depends on microtubules, and could be prevented by the microtubule-destabilizing agent in *M. oryzae* (Zheng et al., 2015). When treating with benomyl, the *MoMih1* mutant showed both obviously increased sensitivity to fungicide and convergent localization of MoVps26-eGFP nearby the hyphal tips, compared with that of *KU80* (**Figures 9C, D, E**). Besides, the ratio of the hyphae containing the swollen tips has remarkably increased in the *MoMih1* mutants (**Figure 9F**), compared with control strain.

### MoMih1 regulates the MoCdc28 phosphorylation in *M. oryzae*

**Figure 10:**
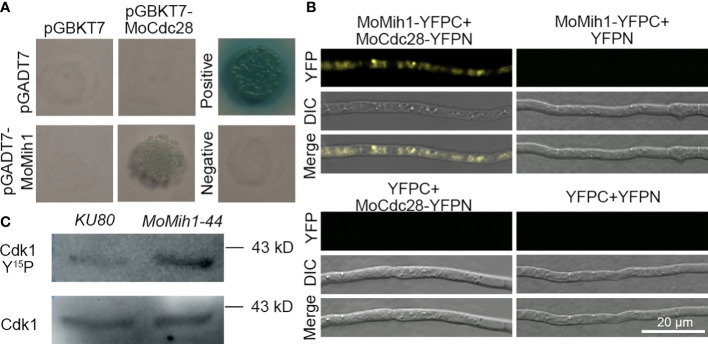
MoMih1 physically interacts with MoCdc28 and regulates the Cdk1 phosphorylation in *M. oryzae* **(A)** Yeast two-hybrid assay. The coding sequences of MoMih1 and MoCdc28 were inserted into the vector pGADT7 and pGBKT7, respectively, and then co-transformed into yeast strain AH109. Yeast cells co-transformed with pGADT7/pGBKT7-MoCdc28, pGADT7-MoMih1/pGBKT7, pGADT7-T/pGBKT7-53, and pGADT7-T/pGBKT7-Lam were used as control, respectively. **(B)** Detection of interaction between MoMih1 and MoCdc28 by bimolecular fluorescence complementation (BiFC) assay. A pair of MoCdc28-^N^YFP and ^C^YFP-MoMih1 constructs were co-transformed into wild type strain Guy11. Construct pairs of MoCdc28-^N^YFP and ^C^YFP, ^C^YFP-MoMih1 and ^N^YFP, and ^N^YFP and ^C^YFP were co-transformed into Guy11 to serve as negative controls. YFP signals were detected under the CLSM. **(C)** Detection of the Cdk1 phosphorylation in the *MoMih1* mutant. Western blot was carried out to detect Cdk1 Y^15^P (for phosphorylation signal) and Cdk1 in protein extracts using the anti-phospho-Cdc2 (Tyr15) and anti-Cdc2 antibodies, respectively. Note that the phosphorylation signal was intensified in *MoMih1* deletion mutant.

In *S. cerevisiae*, protein tyrosine phosphatase Mih1 control the cell cycle progress through affecting the phosphorylation levels of Cdc28. In *M. oryzae*, a putative catalytic subunit of Cdk1 protein named as MoCdc28 (MGG_01362) was identified to directly interact with MoMih1 based on yeast two-hybrid (Y2H) and BiFC assay (**Figures 10A, B**). Moreover, western blot analysis with anti-phospho-Cdk1 antibody also revealed that the Cdk1 phosphorylation signal in the *MoMih1* mutant was much stronger than that in the *KU80* (**Figure 10C**), suggesting an essential role of MoMih1 in removing the phosphate group on MoCdc28 during fungal development in *M. oryzae*.

### MoMih1 is required for nuclear segregation in *M. oryzae*

**Figure 11:**
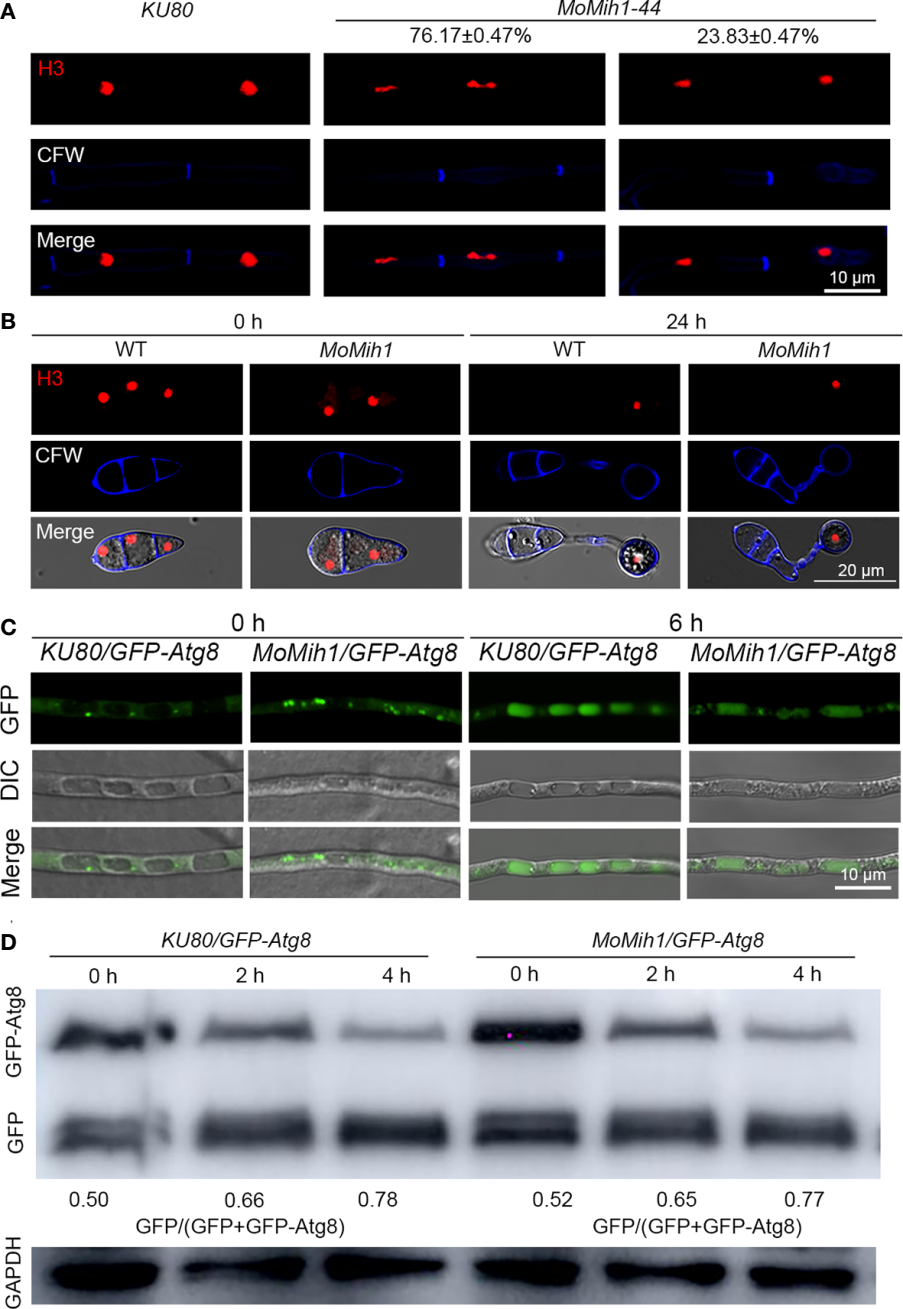
*MoMih1* is required for nuclear division but not non-selective autophagy in *M. oryzae.* **(A)** Nuclear division in the hyphae of *MoMih1* mutant. Hyphae of the *KU80* and *MoMih1* mutant that respectively expressed H3-RFP were stained with CFW and examined by CLSM. Bar = 10 μm. The proportion of each type of fungal cells was indicated in the figure. **(B)** Nuclear distribution during the appressorium development in *MoMih1* mutant. Conidia of *KU80* and *MoMih1* mutant that respectively expressed H3-RFP were inoculated on hydrophobic coverslip for 0 or 24 h, stained with CFW, and examined by CLSM. Bar = 20 μm. **(C)** Localization of eGFP-MoAtg8 in the *MoMih1-44* mutant. Both the parental strain *KU80* and *MoMih1-44* mutant were grown in liquid CM for 36 h, transferred to SD-N medium for 4 h and then examined by CLSM. Bar = 10 μm. **(D)** Immunoblot to detect the degradation of eGFP-MoAtg8. All tested strain were under the treatment of nitrogen starvation for 2 and 4 h, and then immunoblotted with anti-GFP antibody to analyze the breakdown of eGFP-MoAtg8 in the *KU80* and *MoMih1* mutant. The ratio of eGFP with eGFP+eGFP-MoAtg8 was calculated.

To determine whether MoMih1 affects the cell cycle of *M. oryzae*, nuclear segregation was examined in the strains *KU80*-*H3*-*mRFP* and *MoMih1*-*H3*-*mRFP*. Most of the nuclei showed delayed separation in the mycelium of *MoMih1* mutant, compared with *KU80* (**Figure 11A**). However, nuclear segregation seems normally proceed in the *MoMih1* mutant during the appressorium formation, which is similar with the wild type strain *KU80*-*H3*-*mRFP* (**Figure 11B**).

### MoMih1 is dispensable for non-selective autophagy in *M. oryzae*

Autophagy usually synchronized with the cell cycle in *M. oryzae*, to test whether MoMih1 regulates the cell cycle *via* affecting the fungal autophagy, the autophagy was compared between the *MoMih1* mutant and *KU80*. When grown in CM, both the *KU80*-*eGFP*-*Atg8* and *MoMih1*-*eGFP*-*Atg8* strains, which expressed eGFP-MoAtg8 fusion protein, showed similar amount of autophagosomes within hyphae (**Figure 11C**). After transferring into minimum medium minus nitrogen (MM-N), interestingly, the *MoMih1* mutant accumulated similar autophagosomes to that of in wild-type *KU80*-*eGFP*-*Atg8* (**Figure 11C**). Western blot showed that the non-selective autophagy in *KU80*-*eGFP*-*Atg8* strain was comparable with *MoMih1*-*eGFP*-*Atg8* strain (**Figure 11D**), suggesting that MoMih1 is dispensable for non-selective autophagy in *M. oryzae*.

## Discussion

Successful proceeding of mitosis is necessary for the growth and development of eukaryotes (Hindley et al., 1987). In *M. oryzae*, cell cycle regulates the appressorium morphology and plant infection, and the completion of mitosis is critical for the full virulence of this fungus (Oses-Ruiz et al., 2017). In *M. oryzae* genome, a series of cell cycle regulators that is homologous to proteins involved in cell cycle regulation in *S. cerevisiae*, were identified (Oses-Ruiz and Talbot, 2017), and some of them have been verified to participate in the growth, development, sporulation and pathogenicity of this fungus (Saunders et al., 2010; Yue et al., 2017; Li et al., 2018), suggesting the essential roles of the cell cycle regulation during fungal development and plant infection of *M. oryzae*. In *S. pombe*, the dual-specificity phosphatase Cdc25 work together with Wee1 kinase to regulate the phosphorylation of Cdk1 to control the proceeding of mitosis, and the dephosphorylation of Cdk1 by Cdc25 promotes the cells to enter mitosis (Dunphy, 1994). In *S. cerevisiae*, deletion of *Mih1*, a homolog of Cdc25 in fission yeast, results in abnormally enlarging cells and moderately delayed mitotic process (Pal et al., 2008). Besides, Cdc25 and its homologs play also important roles in fungal growth and pathogenicity in many other filamentous fungi, and for example, the disruption of Cdc25 could incur retarded growth, diminished sporulation, and attenuated ability to infect the hosts by *Beauveria bassiana* and *U. maydis* (Sgarlata and Perez-Martin, 2005; Qiu et al., 2015), implying the critical conserved role of this Cdc25 protein during the growth and pathogenesis of those fungi. In *M. oryzae*, a dual-specificity phosphatase name as MoMih1 has been identified in the fungal genome, however, the underlying mechanism regulated by this cell cycle protein has yet to be understood till now. In this study, we found that MoMih1 is conserved in structures that are unique to Cdc25 and Mih1 phosphatases in *S. pombe* and *S. cerevisiae*, respectively. MoMih1 is required for normal mitosis and fungal development in *M. oryzae*, and deletion of *MoMih1* lead to decreased activity of extracellular enzyme and ability to scavenge active oxygen of *M. oryzae*. These results indicate that MoMih1 plays an important role in determining the fungal morphogenesis and virulence of *M. oryzae*.

The hyphae of the filamentous fungal pathogens are highly polarized cells, and their polar growth at the hyphal tip is a prerequisite for vegetative growth, development and plant infection of those pathogens (Steinberg, 2007; Guo et al., 2015). In fungal pathogens, polarized growth depends on a specialized protein subcomplex called polarisome (Harris et al., 2005). The accurate localization of Spa2, a central component of polarisome, is required for establishment of proper hyphae polarized growth in filamentous fungal pathogens (Meyer et al., 2008; Li et al., 2014; Cao et al., 2021; Zheng et al., 2021b; Zhu et al., 2022). In this study, approximate 40% of the vegetative hyphae of the MoMih1 showed abnormally swollen tips, and the central localization of MoSpa2-eGFP observed in the polarisome of *KU80* was completely abolished in the swollen hyphae tips or asymmetrically localized in the twisted hyphae tips of *MoMih1* mutant. In view of the critical role of MoMih1 in protein dephosphorylation, and improper localization of Spa2 homologs to polarisome resulted in defective hyphal tip growth in *M. oryzae* and other phytopathogens (Li et al., 2014; Cao et al., 2021; Zhu et al., 2022), we speculate that MoMih1 might regulate polarized growth by dephosphorylation of certain components of the polarisome, such as MoSpa2, during fungal development in *M. oryzae*. Besides, continuous polarized tip growth also requires the constant delivery of cargo to the cell apex (Pantazopoulou et al., 2014). MoVps26, a subunit of retromer subcomplex that participate in intracellular transport of secretory vesicles cargo (Zheng et al., 2015), showed punctate structures at or near the vacuolar of *KU80*, but dispersed, cytoplasmic signals in the *MoMih1* mutant, implying an essential role of MoMih1 in establishment of cargo selection complex of the retromer in *M. oryzae*. The treatment of benomyl resulted in remarkably convergent localization of MoVps26-eGFP nearby the hyphal tips and increased ratio of swollen hyphal tips in *MoMih1* mutants, further suggesting that MoMih1 might affect the polarized tip growth by destabilizing the microtubule cytoskeleton in the hyphae of *M. oryzae*. Based above, the abolishment of MoSpa2 and disordered localization of MoVps26 that affected by MoMih1 might be the reason for the abnormal polarized tip growth in *M. oryzae*.

The massive formation and diffusion of asexual spore directly determines the epidemic degree of the rice blast in the disease cycle (Zhang et al., 2016). Young conidia are produced on the tip of conidiophores differentiated from the fluffy arial hyphae in an axial manner (Liu et al., 2010). The fluffy growth of arial hyphae is proportional to the development of conidiophores, and essential for asexual sporulation of *M. oryzae* (Jeon et al., 2008; Guo et al., 2017; Matheis et al., 2017). In *M. oryzae*, deletion of the *MoMih1* produced less arial hyphae, and significantly reduced conidiophores and conidia, suggesting a critical role of MoMih1 in determining the asexual development in the blast fungus. The significant downregulation of two transcription factors MoFLBC and MoFLBD, which are essential for aerial hyphae development and spore formation (Matheis et al., 2017), respectively, might be partially responsible for defects of conidiophore development and conidial formation in *MoMih1* mutant. The MoACR1, a stage-specific negative regulator for conidiation, is essential for the establishment of sympodial pattern of spore formation, and a mutation of this protein lead to produce head-to-tail conidia in *M. oryzae* (Lau and Hamer, 1998). Besides, blocking of *MoSsadh* also completely abolished the conidiation due to the loss of conidiophore development in *M. oryzae* (Guo et al., 2011). We here found that the transcription level of those two genes significantly decreased in the *MoMih1* mutant, further suggesting an important role of MoMih1 in conidiophore development. Taken together, our results implied that MoMih1 might function as a key upstream regulator to stimulate the transcription of conidiation related genes during asexual development of *M. oryzae*.

The scavenging of ROS at the infection site of the plants is a prerequisite for the successful fungal penetration during plant-microbe interaction (Molina and Kahmann, 2007; Chi et al., 2009; Guo et al., 2010; Guo et al., 2011; Zhang et al., 2021). In *M. oryzae*, the basic leucine zipper (bZIP) family transcriptional factors MoAP1 and Moatf1 are two important regulators in response to oxidative stressors, and deletion either of them resulted in attenuation of degrading the host-derived ROS, and thus reduced the pathogenicity of the mutants (Guo et al., 2010; Guo et al., 2011). In this study, deletion of *MoMih1* attenuated the fungal virulence, with both decreased penetration and retarded invasive hyphae extension during plant infection. The *MoMih1* mutant was more sensitive to hydrogen peroxide, and almost lost the secretion of extracellular laccases and peroxidases that help to scavenge the host-derived ROS. Since ROS generally acts as the second messenger that activate the defense pathway during plant microbe interactions (Molina and Kahmann, 2007; Guo et al., 2010; Guo et al., 2011), we speculated that MoMih1 may participate in fungal pathogenicity by regulating the extracellular enzyme activity to detoxify host-derived ROS and then facilitated the fungal penetration and invasive hyphae extension in *M. oryzae*.

The plant infection of *M. oryzae* is mediated by development of a functional appressorium (Wilson and Talbot, 2009; Zhang et al., 2011; Ryder et al., 2019). The proceeding of cell cycle is critical for the formation of appressorium and secondary infection hyphae (Saunders et al., 2010; Oses-Ruiz et al., 2017). In *S. cerevisiae*, the checkpoint that inhibits anaphase onset mainly depends on the activity of Cdk1, and the Mih1 has been proved to remove the phosphate group of Cdk1^Y19^, which as a result activated the Cdk1 activity, and promote the transition of mitosis in *S. cerevisiae* (Pal et al., 2008). In this study, MoMih1 directly interacted with the Cdk1 homologue MoCdc28 *in vivo*, and deletion of *MoMih1* resulted in incremental numbers of nucleus in many hyphal cell compartments of the fungus and significantly increased phosphorylation level of MoCdc28^Y15^ in *MoMih1* mutant, confirmed that MoMih1 functioned as important cell cycle regulator to proceed the mitosis during development of *M. oryzae*. This finding agrees with the role of Cdc25 in *S. pombe* and *U. maydis*, in which the deletion of Cdc25 strongly blocked the initiation of M phase, further suggesting a conserved role of Cdc25 tyrosine phosphatases in controlling the cell division in diverse fungi (Sgarlata and Perez-Martin, 2005). Interestingly, nuclear division arrest was not identified during appressorium development, we attributed this to the rapid mitosis whose duration time is too short to be captured during conidia germination in *MoMih1* mutant. In *S. cerevisiae*, Mih1 localized to the nucleus and cytoplasm through the mechanism of nucleocytoplasmic shuttle, and in most situation functioned in the nucleus (Yano et al., 2013). In this study, MoMih1 was localized to both the nucleus and cytoplasm at mycelial stage, whereas only located in the cytoplasm at conidial stage, indicating the conserved role of MoMih1 during fungal development, and this spatial expression difference at diverse stages might reflect a critical role of MoMih1 in determining polar growth, cargo transport and full virulence of *M. oryzae*.

In conclusion, this study revealed the role of *MoMih1* in the development of *M. oryzae*, which laid a foundation for further study on the role of *MoMih1* in the cell cycle of *M. oryzae*.

## Data availability statement

The original contributions presented in the study are included in the article/**Supplementary Material**. Further inquiries can be directed to the corresponding author.

## Author contributions

MG conceived the experiments. SL and XG performed the experiments. MG and SL wrote the manuscript. JM and SW helped to analyze the data and revise the manuscript. All authors contributed to the article and approved the submitted version.

## Supplementary material

Supplementary Figure 1 Validation of *MoMih1* deletion in *M. oryzae*. **(A)**. The gene deletion strategy for generating the *MoMih1* mutants. **(B)**. The *M. oryzae MoMih1* mutants were validated by PCR. The genomic DNA was extracted from *MoMih1-41* and *MoMih1-44*, and verfied using the primers of P1/P2 or P3/P4 by PCR. The *MoMih1* deletion mutants can amplify 1.485 and 1.486 kb fragments using the primers of P1/P2 or P3/P4. **(C)**. The *M. oryzae MoMih1* mutants were validated by RT-PCR. The total RNA was extracted from *KU80*, *MoMih1-41*, *MoMih1-44*, and *MoMih1*c, reversely transcribed, and verfied using the primer pairs to detect *MoMih1* (MGG_07734) and *Actin* (MGG_03892). (D, E). Southern blot to validate the deletion of *MoMih1* mutants (*MoMih1-41*, *MoMih1-44*). To validate the deletion of *MoMih1* in *M. oryzae*, genomic DNA of *KU80* and *MoMih1* deletion mutants were digested with *Sph*I and hybridized with *MoMih1*-LF probe; To validate the copy number of *HPH* in *MoMih1* deletion mutants, genomic DNA of *KU80* and *MoMih1* deletion mutants were digested with *Hin*dIII, and hybridized with the *HPH* probe.

Supplementary Figure 2 The Validation of *MoGin4* and *MoCyc2* deletion in *M. oryzae*. **(A)**. The *M. oryzae MoGin4* mutants were validated by PCR. The genomic DNA was extracted from *MoGin4-2* and *MoGin4-5* mutants, and detected using the primers GP1/P2 and P3/GP4 by PCR. The *MoGin4* deletion mutants can amplify 1.601and 1.504 kb fragments by the primers GP1/P2 and P3/GP4. **(B)**. The *M. oryzae MoGin4* mutants were validated by RT-PCR. The total RNA was extracted from Guy11, *MoGin4-2*, and *MoGin4-5*, reversely transcribed, and verfied by the primers MoGin4-RT-1 and MoGin4-RT-2 to detect the deltion of *MoGin4* (MGG_02810). **(C)**. The *M. oryzae MoCyc2* mutants were validated by PCR. The genomic DNA was extracted from *MoCyc2-4* and *MoCyc2-14*, and validated using the primers CP1/P2 and P3/CP4 by PCR. The *MoCyc2* deletion mutants can amplify 1.586 and 1.604 kb fragments, respetively. **(D)**. The *MoCyc2* mutants were validated by RT-PCR. The total RNA of Guy11, *MoCyc2-4*, and *MoCyc2-14* was respetively extracted, reversely transcribed, and verfied by using the primer pairs MoCyc2-RT-1 and MoCyc2-RT-2 to detect the deletion of *MoCyc2* (MGG_07065).

Supplementary Figure 3 Bioinformatic analysis of MoMih1 and Cdc25 homologues. Bioinformatic analysis revealed that MoMih1 contains a conserved HCXXXXXR sequence and DCR motif unique to Cdc25 and Mih1 phosphatases in *S. pombe* and *S. cerevisiae*, respectively. The conserved DCR motif (aa 392-394) and the HCXXXXXR consensus sequence (aa 434-441) are marked with the black frame.

Supplementary Figure 4 The subcellular localization of MoMih1 in *M. oryzae*. The conidia **(A)**, mycelia **(B)**, and invasive hyphae **(C)** of the transformants expressing the MoMih1-eGFP fusion protein were examined under a CLSM.

Supplementary Figure 5 The *MoMih1* mutants are hypersensitive to hydrogen peroxide in *M. oryzae*. **(A)**. Mycelia growth under hydrogen peroxide. The *KU80*, *MoMih1* mutants and *MoMih1*c were inoculated on CM with or without 5 mM H_2_O_2_ and cultured at 28 °C for 5 days. **(B)**. Statistical analysis of the inhibition rate under hydrogen peroxide. “**” stands for the significant difference among the tested strains (*p* < 0.01), and error bars represent SD. **(C)**. Laccase activity test. The laccase activity was monitored in CM supplemented with 0.2 mM 2, 2’-azino-di-3-ethylbenzathiazoline-6-sulfonate (ABTS). **(D)**. Measurement of the laccase and peroxidase activity. **(E)**. Transcription of peroxidases and laccases encoding genes. The expression of putative peroxidases and laccases encoding genes was examined by qRT-PCR in the *KU80* and *MoMih1* mutants.

Supplementary Figure 6 MoMih1 is required for the hydrophobicity of *M. oryzae*. **(A)**. Hydrophobicity assay for the *MoMih1* mutants. Drops of water were inoculated on the colony surfaces of the tested strains. An easily-wettable phenotype of *MoMih1* mutants was observed and photographed at 24 hpi. **(B)**. The transcriptional levels of hydrophobin genes. The expression of four hydrophobin genes, such as MGG_09134, MGG_10105, *MoMPG1*, and *MoMHP1*, was significantly reduced in the *MoMih1* mutant. “**” mean significant difference in gene expression (*p*< 0.01), and error bars represent SD.
