# Supplementary figures and images for: MoMih1 is indispensable for asexual development, cell wall integrity, and pathogenicity of Magnaporthe oryzae
Source: Front Plant Sci. 2023 Mar 14;14:1146915. doi: 10.3389/fpls.2023.1146915 (PMC10044144; doi:10.3389/fpls.2023.1146915)

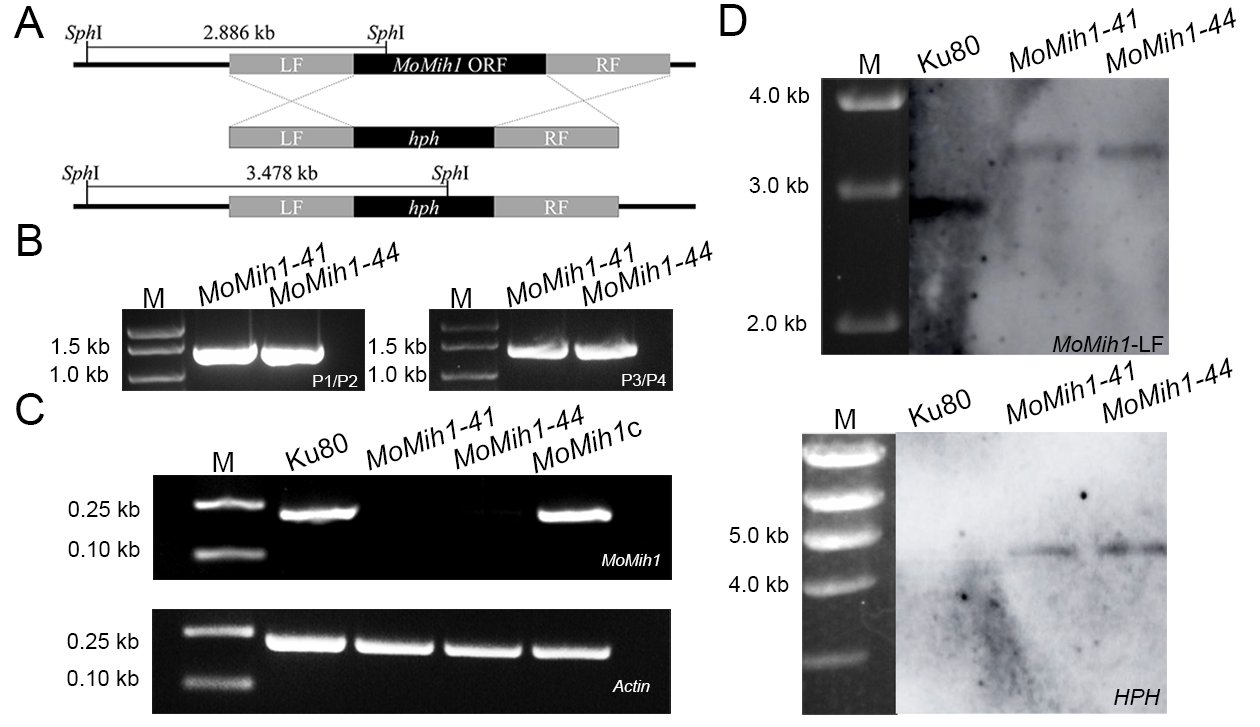

Supplement: Supplementary Figure 1 — Validation of MoMih1 deletion in M. oryzae. (A). The gene deletion strategy for generating the MoMih1 mutants. (B). The M. oryzae MoMih1 mutants were validated by PCR. The genomic DNA was extracted from MoMih1-41 and MoMih1-44, and verfied using the primers of P1/P2 or P3/P4 by PCR. The MoMih1 deletion mutants can amplify 1.485 and 1.486 kb fragments using the primers of P1/P2 or P3/P4. (C). The M. oryzae MoMih1 mutants were validated by RT-PCR. The total RNA was extracted from KU80, MoMih1-41, MoMih1-44, and MoMih1c, reversely transcribed, and verfied using the primer pairs to detect MoMih1 (MGG_07734) and Actin (MGG_03892). (D, E). Southern blot to validate the deletion of MoMih1 mutants (MoMih1-41, MoMih1-44). To validate the deletion of MoMih1 in M. oryzae, genomic DNA of KU80 and MoMih1 deletion mutants were digested with SphI and hybridized with MoMih1-LF probe; To validate the copy number of HPH in MoMih1 deletion mutants, genomic DNA of KU80 and MoMih1 deletion mutants were digested with HindIII, and hybridized with the HPH probe. [file Image_1.tif]

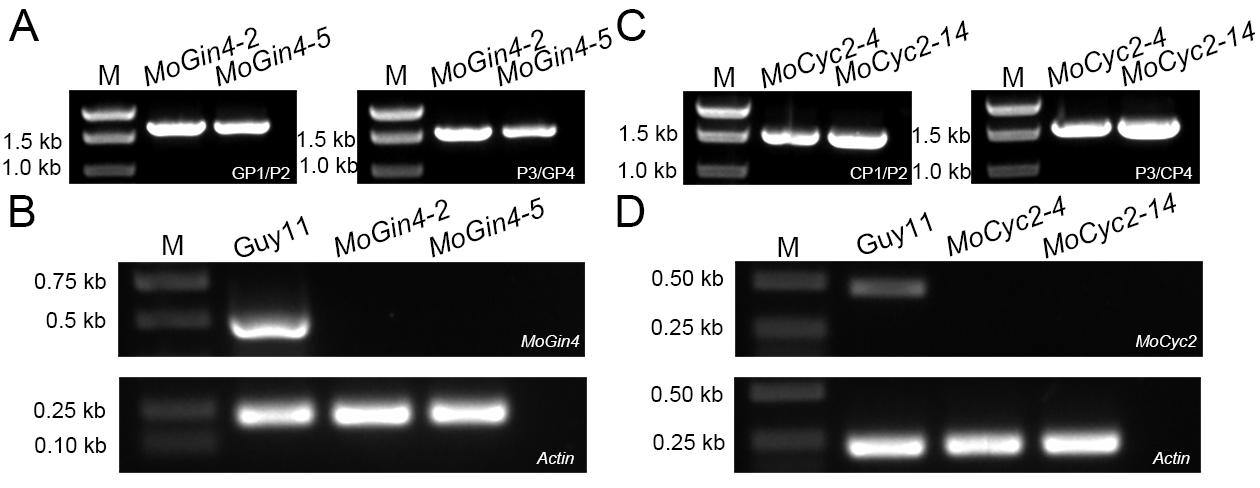

Supplement: Supplementary Figure 2 — The Validation of MoGin4 and MoCyc2 deletion in M. oryzae. (A). The M. oryzae MoGin4 mutants were validated by PCR. The genomic DNA was extracted from MoGin4-2 and MoGin4-5 mutants, and detected using the primers GP1/P2 and P3/GP4 by PCR. The MoGin4 deletion mutants can amplify 1.601and 1.504 kb fragments by the primers GP1/P2 and P3/GP4. (B). The M. oryzae MoGin4 mutants were validated by RT-PCR. The total RNA was extracted from Guy11, MoGin4-2, and MoGin4-5, reversely transcribed, and verfied by the primers MoGin4-RT-1 and MoGin4-RT-2 to detect the deltion of MoGin4 (MGG_02810). (C). The M. oryzae MoCyc2 mutants were validated by PCR. The genomic DNA was extracted from MoCyc2-4 and MoCyc2-14, and validated using the primers CP1/P2 and P3/CP4 by PCR. The MoCyc2 deletion mutants can amplify 1.586 and 1.604 kb fragments, respetively. (D). The MoCyc2 mutants were validated by RT-PCR. The total RNA of Guy11, MoCyc2-4, and MoCyc2-14 was respetively extracted, reversely transcribed, and verfied by using the primer pairs MoCyc2-RT-1 and MoCyc2-RT-2 to detect the deletion of MoCyc2 (MGG_07065). [file Image_2.tif]

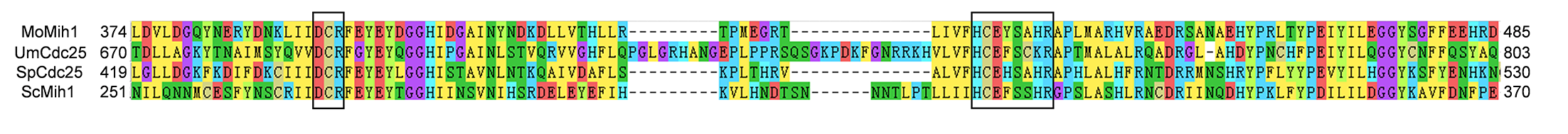

Supplement: Supplementary Figure 3 — Bioinformatic analysis of MoMih1 and Cdc25 homologues. Bioinformatic analysis revealed that MoMih1 contains a conserved HCXXXXXR sequence and DCR motif unique to Cdc25 and Mih1 phosphatases in S. pombe and S. cerevisiae, respectively. The conserved DCR motif (aa 392-394) and the HCXXXXXR consensus sequence (aa 434-441) are marked with the black frame. [file Image_3.tif]

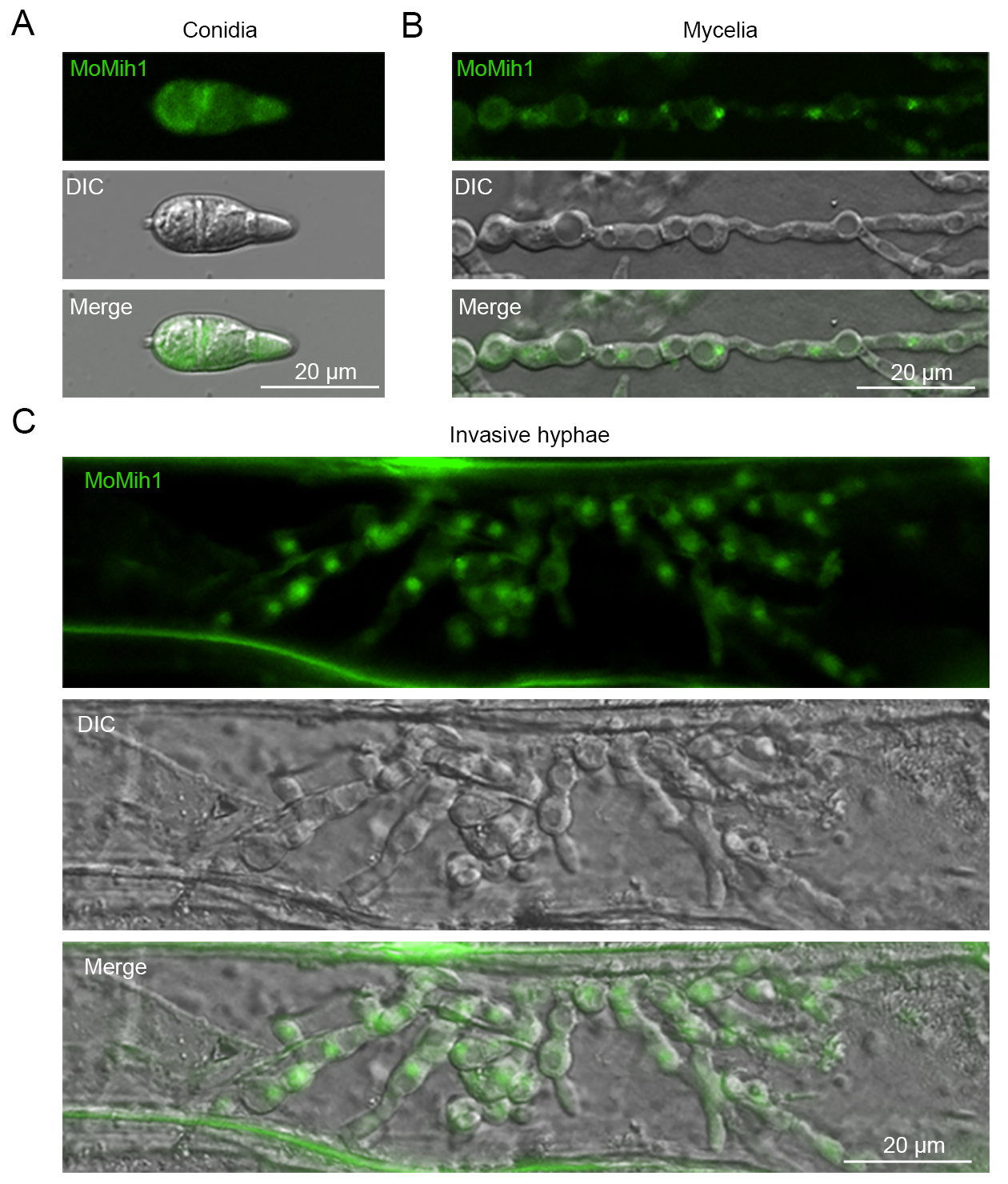

Supplement: Supplementary Figure 4 — The subcellular localization of MoMih1 in M. oryzae. The conidia (A), mycelia (B), and invasive hyphae (C) of the transformants expressing the MoMih1-eGFP fusion protein were examined under a CLSM. [file Image_4.tif]

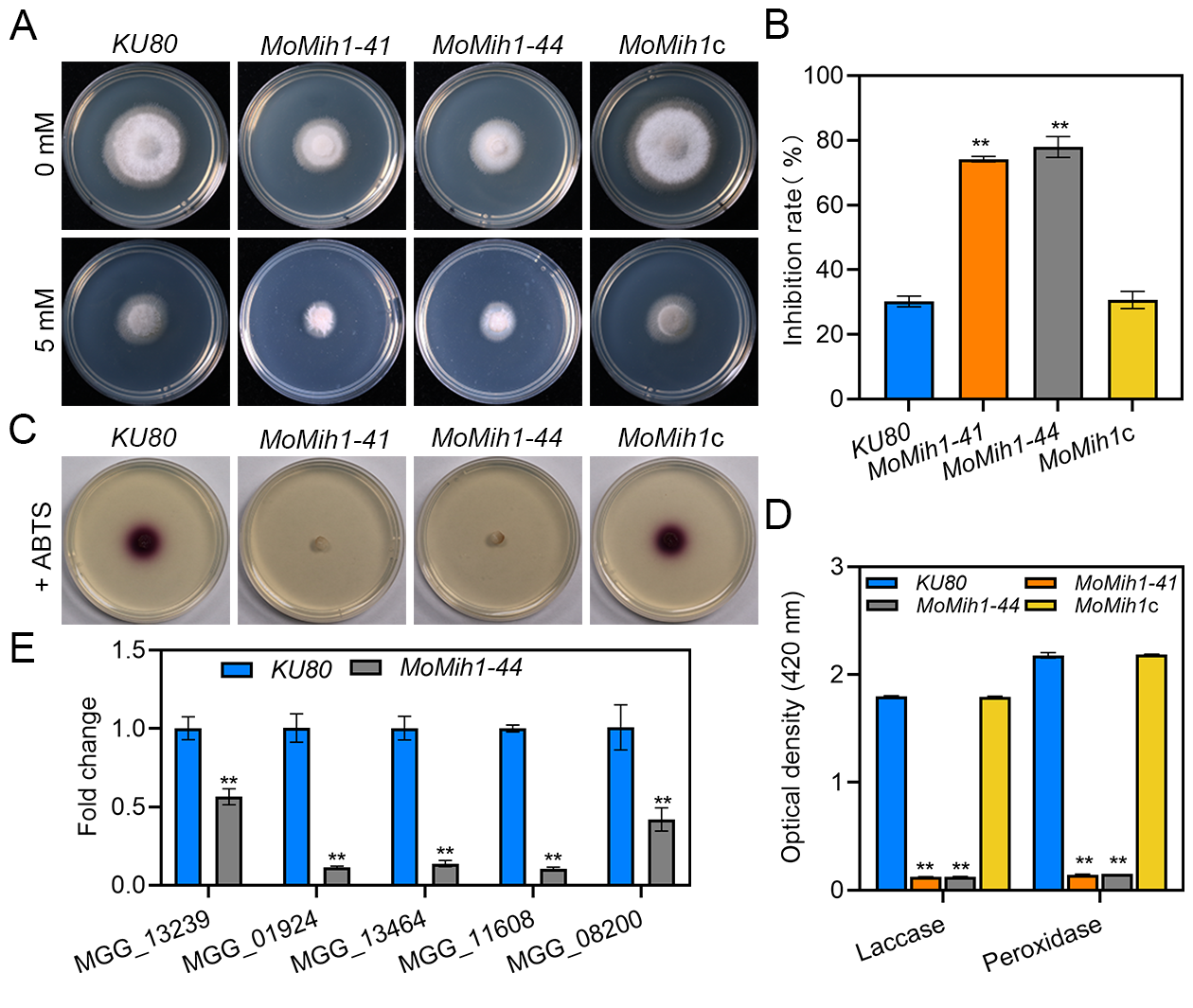

Supplement: Supplementary Figure 5 — The MoMih1 mutants are hypersensitive to hydrogen peroxide in M. oryzae. (A). Mycelia growth under hydrogen peroxide. The KU80, MoMih1 mutants and MoMih1c were inoculated on CM with or without 5 mM H2O2 and cultured at 28 °C for 5 days. (B). Statistical analysis of the inhibition rate under hydrogen peroxide. “**” stands for the significant difference among the tested strains (p < 0.01), and error bars represent SD. (C). Laccase activity test. The laccase activity was monitored in CM supplemented with 0.2 mM 2, 2’-azino-di-3-ethylbenzathiazoline-6-sulfonate (ABTS). (D). Measurement of the laccase and peroxidase activity. (E). Transcription of peroxidases and laccases encoding genes. The expression of putative peroxidases and laccases encoding genes was examined by qRT-PCR in the KU80 and MoMih1 mutants. [file Image_5.tif]

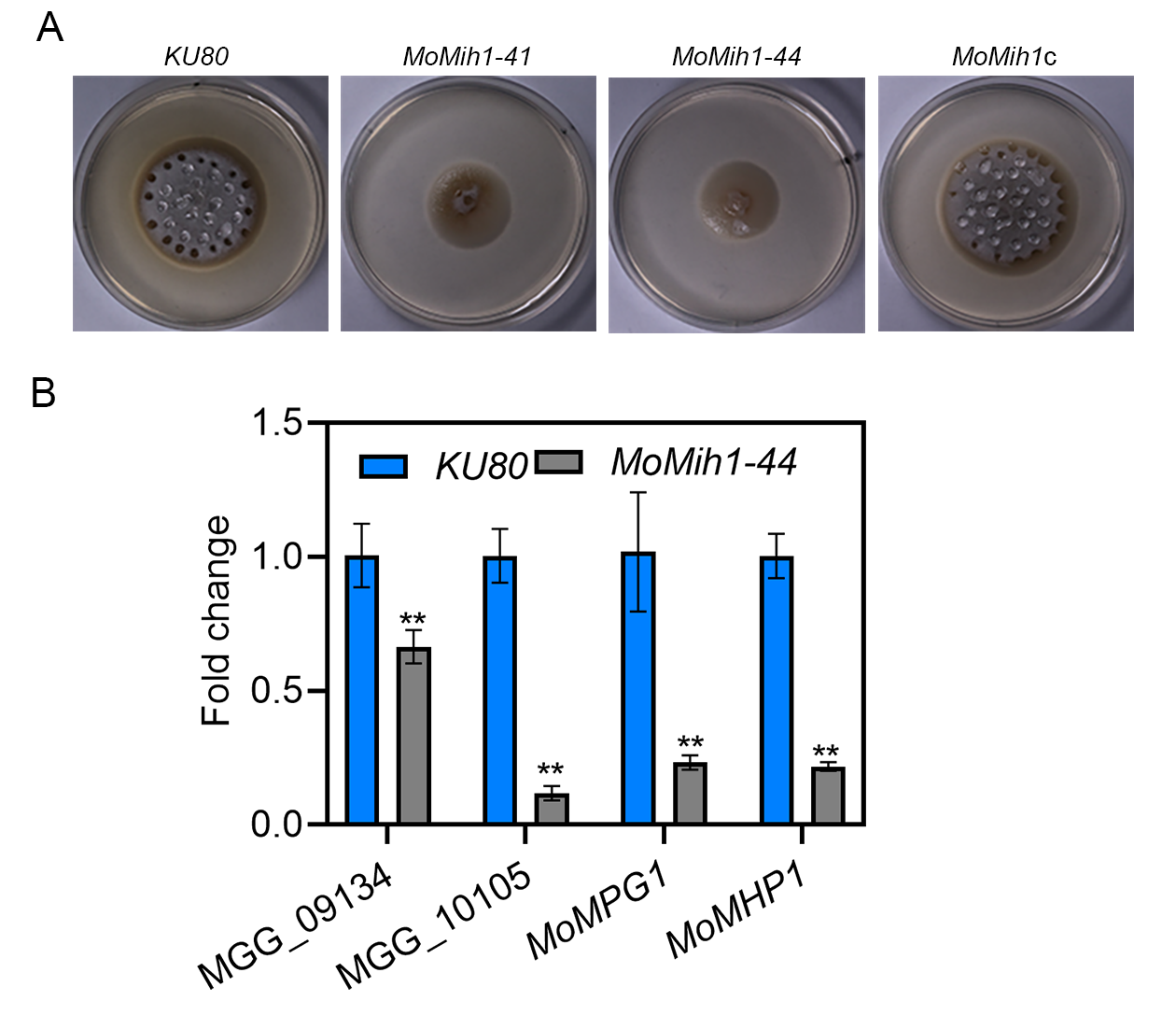

Supplement: Supplementary Figure 6 — MoMih1 is required for the hydrophobicity of M. oryzae. (A). Hydrophobicity assay for the MoMih1 mutants. Drops of water were inoculated on the colony surfaces of the tested strains. An easily-wettable phenotype of MoMih1 mutants was observed and photographed at 24 hpi. (B). The transcriptional levels of hydrophobin genes. The expression of four hydrophobin genes, such as MGG_09134, MGG_10105, MoMPG1, and MoMHP1, was significantly reduced in the MoMih1 mutant. “**” mean significant difference in gene expression (p< 0.01), and error bars represent SD. [file Image_6.tif]
