# Supplementary material for: MoMih1 is indispensable for asexual development, cell wall integrity, and pathogenicity of Magnaporthe oryzae
Source: Front Plant Sci. 2023 Mar 14;14:1146915. doi: 10.3389/fpls.2023.1146915 (PMC10044144; doi:10.3389/fpls.2023.1146915)
Supplement: Supplementary file 7 [file Table_1.docx]

| Table S1 Primers | | |
| --- | --- | --- |
| Primer | Sequence(5＇-3＇) | Use |
| *MoMih1*-1F | GGTACCCGGGGATCCTCTAGAGCTGGATGATGATGTAAGGCAG | Construction of  pKO1B-*MoMih1*-*HPH* |
| *MoMih1*-2R | TTCATTGTTGACCTCCACTAGATTACGGGTGGTTATGCGAG |  |
| *MoMih1*-3F | GCAAAGGAATAGAGTAGATGGACGGACCTCAACTATTCA |  |
| *MoMih1*-4R | ACGACGGCCAGTGCCAAGCTTCCGTTTCTCTTATACTCCTCGCAC |  |
| HPH-F | TAGTGGAGGTCAACAATGAATG |  |
| HPH-R | CATCTACTCTATTCCTTTGCC |  |
| P1 | GAACGACCGATACTGTTGAG | Verification of *MoMih1*, *MoGin4*, *MoCyc2* deletion mutants |
| P2 | AATTTTGTGCTCACCGCCTG |  |
| P3 | TACTCGCCGATAGTGGAAACC |  |
| P4 | GTAGTGACCAGACTGACGAA |  |
| *MoMih1*-probe-F | TTTCTGCTGCTCTCACGGCAA |  |
| *MoMih1*-probe-R | GATTACGGGTGGTTATGCGAG |  |
| *MoMih1*-RT-1 | AACTCCCTCCCTTCCGTTTT |  |
| *MoMih1*-RT-2 | CTGCTACCATTGAGGCTAGA |  |
| GP1 | CGGAGCATCATGTTTGCTTC |  |
| GP4 | CATGACCACTATCCCCAAGTC |  |
| *MoGin4*-RT-1 | CCACCGGAGAAGACTTCGAATAAG |  |
| *MoGin4*-RT-2 | GAACAATGCTGAGGTGGTTACG |  |
| CP1 | GTAAGCGTGTCGCAACCTATC |  |
| CP4 | CAAGGCTCTACGTGGTATTG |  |
| *MoCyc2*-RT-1 | CACTGCCCATTCTTACCGATC |  |
| *MoCyc2*-RT-2 | GTTGATCTCCTCGTACTTGG |  |
| AD-MoMih1-F | GAGTACCCATACGACGTACCAGATTACGCTATGGAGGACTCATCACCATTGGCG | Amplification of  *MoMih1* and *MoCdc28* cDNA in *M. oryzae* |
| AD-MoMih1-R | TCTACGATTCATCTGCAGCTCGAGCTCGATTCAGTACGATGCCATTCTCCTACCA |  |
| BD-MoCdc28-F | AGCTGATCTCAGAGGAGGACCTGATGGAAAACTACCAGAAGCTCGAGAAG |  |
| BD-MoCdc28-R | ACCCGTTTAGAGGCCCCAAGGGGTTATGCTAGCTATCGCCTCGGGGGCGC |  |
| MoRSY1-QF | CGACTCCAAGGACTGGGATA | Amplification of *M. oryzae* melanin  related genes by  qRT-PCR |
| MoRSY1-QR | GTCCTCGGACACCTTCTCC |  |
| MoALB1-QF | GCAATGTCGGTCCCAACTAC |  |
| MoALB1-QR | ATCTCAAAGGCGATGACACC |  |
| MoBUF1-QF | ACGCCGTCTACTCAGGATCA |  |
| MoBUF1-QR | TCTCGCCGTTTGGAATGTAT |  |
| MoHNR1-QF | GCAAACATGAATCCGCTCAAG |  |
| MoHNR1-QR | CGGTCAACTTGATAACTTGGC |  |
| MoChs1-QF | AGGGAGAGACCGATGTTCCT | Amplification of *M. oryzae*  chitin genes by  qRT-PCR |
| MoChs1-QR | AGCTGGACGTGGAAGAAGAA |  |
| MoChs2-QF | CACCGCCAACATGTATCTGG |  |
| MoChs2-QR | TTCAGCCAACGACGTCTTTG |  |
| MoChs3-QF | CGACCAGCTTCAACTTCACA |  |
| MoChs3-QR | GGAGTCTGAGCTTCGTTTGG |  |
| MoChs4-QF | TCCTGATGTCGTTCTTGCAG |  |
| MoChs4-QR | GATCTCAGGGTCCTTCACCA |  |
| MoChs5-QF | CTAGTGTGGGCCATCACCTT |  |
| MoChs5-QR | CAAACGGCCAAGGAAGATAA |  |
| MoChs6-QF | TATGCGCTACGATGACAAGC |  |
| MoChs6-QR | CGAGTAAACCTTGCCCATGT |  |
| MoChs7-QF | AAACTCGAGGGACATGTTGG |  |
| MoChs7-QR | CCTCCTGAACGCAGAGAAAC |  |
| *MoMPG1*-QF | AAGGTCGTCTCTTGCTGCAA | Amplification of  hydrophobic genes in  *M. oryzae* by  qRT-PCR |
| *MoMPG1*-QR | GGATGTTGACCAGACCAATC |  |
| *MoMHP1*-QF | GGTTCCCTACACTCCCTGCT |  |
| *MoMHP1*-QR | GCAGAGGATACCCTGGTCAA |  |
| MGG_09134-QF | AGATCAAGGCCCTCATCGTC |  |
| MGG_09134-QR | GATGTCAGTGGCACAGCACT |  |
| MGG_10105-QF | CGGCAGCGGAGACTATGA |  |
| MGG_10105-QR | CGCAAATGTCGGTGAAGC |  |
| MoSsadh-QF | CCTACGCAGCCAACTTTTTC | Amplification of *M. oryzae* conidiation  related genes by  qRT-PCR |
| MoSsadh-QR | AGGACCGATCTTACGTGTGC |  |
| MoACR1-QF | GAGCAACTCGATCTGCATCA |  |
| MoACR1-QR | AACTGTGATGGGCTTGAACC |  |
| MoFLUG-QF | CGTTCTTCCTCGTCAGCTTT |  |
| MoFLUG-QR | GGACGATGGTGTCCTGAACT |  |
| MoFLBC-QF | GGATACGCAGAAACCCCATA |  |
| MoFLBC-QR | ACTCCTCTCGTCTTGGTGGA |  |
| MoFLBD-QF | CTAGACAAAGCGCATCACGA |  |
| MoFLBD-QR | GGCTTGAGGTTCTGGTGGTA |  |
| MGG_13239-QF | TTGATGTGATTCGCGACGTC | Amplification of  *M. oryzae* laccase and  peroxidase genes by  qRT-PCR |
| MGG_13239-QF | GAGCGGTACATTTCGTGGTC |  |
| MGG_01924-QF | TCCCTGGACTGCTCAAGTCT |  |
| MGG_01924-QR | GCTGTCAAGAGGACGGTAGC |  |
| MGG_13464-QF | CTTCCAGTACGAGGGTGCT |  |
| MGG_13464-QR | TGTTGACGGTCCAGTAGACG |  |
| MGG_11608-QF | GGAGTACTGGCTCTCCATCG |  |
| MGG_11608-QR | TTGGTCTGCATGTTGTTGGT |  |
| MGG_08200-QF | GGATCCCTCATTGCGTTGAC |  |
| MGG_08200-QR | TCACCGAGTATCTGGATGGC |  |
| *Atg8*-F | TCACTCTCGGCATGGACGAGCTGTACAAGATGCGCTCCAAGTTCAAGGAC | Construction of  pMOC^H245Y^-*eGFP*-*Atg8* |
| *Atg8*-R | GCATAAATGGGTGGAGATGCGTTCACTCACTCGACTTCCTCAAACAGGTC |  |
| *H3*-F | AAACTTCGTCGGTTTCTAGGTCACCCTTGCCGCGTCCAGTCATGTTGATT | Construction of  pMOC^H245Y^-*H3*-*mRFP* |
| *H3*-R | TCCTTGATGACGTCCTCGGAGGAGGCCATGTTGCGCTCGCCGCGAA |  |
| *mRFP*-F | ATGGCCTCCTCCGAGGACGT |  |
| *mRFP*-R | CCATGCATAAATGGGTGGAGATGCGTTCACTTAGGCGCCGGTGGAGTGGC |  |
| *Mih1*pF | AAATCGTGGTTCTCATCACCATCACCATCAGGCCCTTGCTCAGAAATTCA | Construction of  pYF11-*MoMih1* and pYF11-  RP27-*MoMih1* |
| *Mih1*pR | CCCGGTGAACAGCTCCTCGCCCTTGCTCACGTACGATGCCATTCTCCTAC |  |
| RP27*Mih1*pF | AAATCGTGGTTCTCATCACCATCACCATCAATGGAGGACTCATCACCATT |  |
| pHZ68Mih1F | CGACTCACTATAGGGCGAATTGGGTACTCAAATTG | BiFC assay |
| pHZ68Mih1R | GTTCGGGATCTTGCAGGCCGGGCGGTACGATGCCATTCTCCTAC |  |
| pHZ65Cdc28F | CACTATAGGGCGAATTGGGTACTCAAATTGCGCTGAGATCTCTGTGAATG |  |
| pHZ65Cdc28R | GCTCACCATCGTGGCGATGGAGCGTCGCCTCGGGGGCGCTGGGGT |  |
|  |  |  |
| Vps26pF | ATCGTGGTTCTCATCACCATCACCATCAATGTCCTACTTCTTCTCGACCC | Construction of  pYF11-*MoVps26* |
| Vps26pR | TGAACAGCTCCTCGCCCTTGCTCACCGCAGGGACCGCCTGTATCCTACTC |  |
| Spa2p1F | AAATCGTGGTTCTCATCACCATCACCATCAATGAACGCTGCTGCCACC | Construction of  pYF11-*MoSpa2* |
| Spa2p1R | CCATTCTTGTGCCTCGGCCTC |  |
| Spa2p2F | GATCTTCGCAGGCTGCTCAGG |  |
| Spa2p2R | CCCGGTGAACAGCTCCTCGCCCTTGCTCACTTATGAAAAGTCATCACCACCACCAGGAC |  |
